# Design and development of ‘Helder in Gesprek’: A tool to support person-centred communication in memory clinics

**DOI:** 10.1177/20552076251412631

**Published:** 2026-01-20

**Authors:** Tanja J de Rijke, Kyra KM Kaijser, Dianne Vasseur, Hilal Tasköprü, Lotte Huisman, Aniek M van Gils, Vera Otten, Carolien Smits, Cynthia S Hofman, Minke Kooistra, Ellen MA Smets, Thomas Engelsma, Leonie NC Visser

**Affiliations:** 1 Department of Medical Psychology, 26066 Amsterdam UMC Location University of Amsterdam, Amsterdam, The Netherlands; 2 1229 Amsterdam Public Health Research Institute, Digital Health/Personalised Medicine/Quality of Care/Aging & Later Life, Amsterdam, The Netherlands; 3 Amsterdam UMC, Location Vrije Universiteit Amsterdam, Alzheimer Center Amsterdam, Amsterdam, The Netherlands; 4 601873 Amsterdam Neuroscience, Neurodegeneration, Amsterdam, The Netherlands; 5 Vilans, 99405 The Dutch National Centre of Expertise for Care and Support, Utrecht, The Netherlands; 6 497785 Alzheimer Nederland, Amersfoort, The Netherlands; 7 Pharos, The Dutch Centre of Expertise on Health Disparities, Utrecht, The Netherlands; 8 Department of Medical Informatics, eHealth Living & Learning Lab Amsterdam, Amsterdam UMC location University of Amsterdam, Amsterdam, The Netherlands; 9 Division of Clinical Geriatrics, Center for Alzheimer Research, Department of Neurobiology, Care Sciences and Society, 27106 Karolinska Institutet, Stockholm, Sweden; 10 Department of Bioethics & Health Humanities, UMC Utrecht, Julius Center, Utrecht, The Netherlands

**Keywords:** Memory clinic, co-design, usability, UX, development, patient–clinician communication, design research, eHealth, mHealth, Alzheimer

## Abstract

**Objective:**

Person-centred communication in memory clinics is essential, but often not optimal. This study aimed to develop a solution that supports people with cognitive complaints in expressing their needs and preferences during memory clinic consultations.

**Methods:**

Following a human-centred design approach, co-researchers (n = 4 people with dementia) identified a problem statement. This problem was confirmed and elaborated upon via a questionnaire (n = 25) and focus group (n = 18) for triangulation purposes, and in co-design sessions with people with cognitive complaints (n = 3), care partners (n = 2), and clinicians (n = 3). These sessions informed prototype development in collaboration with a design agency. Usability and User eXperience (UX) testing were conducted with people with cognitive complaints (n = 30), care partners (n = 4), and clinicians (n = 17) via think-aloud sessions, interviews, questionnaires, and focus groups.

**Results:**

Co-researchers emphasized the importance of clinicians gaining a holistic understanding of someone's life and circumstances, which was confirmed in the ‘triangulation’ questionnaire, focus group, and co-design sessions. Co-design resulted in a digital and analogue prototype of ‘Helder in Gesprek’ (‘Clear in Conversation’), a tool to assist people with cognitive complaints in reflecting on what they wish to share with their clinician and facilitate communication during consultations. Usability testing revealed a generally positive attitude toward the prototypes, while also identifying areas for improvement, such as navigation, system feedback, understandability, distinguishable elements, and cognitive overload.

**Conclusion:**

Our human-centred design approach informed the design and development of two prototypes of ‘Helder in Gesprek’. Usability and UX testing provide directions for re-design and feasibility testing in a real-world setting.

## Introduction

Worldwide, the number of people with dementia is predicted to rise from 50 to 152 million by 2050 due to ageing populations and the lack of effective preventative and/or therapeutic options.^1^ The increasing prevalence raises the demand for dementia-related healthcare, for example, in memory clinics.^2^ Memory clinics are multidisciplinary clinics aiming for a timely diagnosis and support for people with cognitive complaints.^3^ People visiting the memory clinic comprise people with dementia, people with mild cognitive impairment (MCI), in which cognitive decline can be clinically observed, and people with subjective cognitive decline (SCD), who experience cognitive decline without objective clinical cognitive decline.^4^

Due to recent developments regarding dementia prevention, prediction, and diagnosis, more care-related decisions are available for people with cognitive complaints. For example, whether or not to choose between potentially available treatment options, which may have adverse effects, or whether to undergo certain tests to determine dementia risk, such as an amyloid PET scan. People must weigh up the advantages and disadvantages of the available options. The implications of an option can be impacted by their biomedical make-up, such as the underlying pathology, or by their personal preferences, needs, and circumstances. In that regard, a person-centred care approach is necessary, in which people with cognitive complaints are considered to be the expert in their life and should be able to have a say or even lead the direction of their care.^5^ A purely medical understanding of dementia is refuted by the person-centred care approach, which contends that health, psychology, and the social environment all interact in the case of dementia.^6,7^ Prior studies conducted in several disciplines within the Alzheimer's field^8–16^ and among individuals with cognitive complaints or dementia^7,^^17–19^ have emphasized the necessity of such a person-centred care approach.

Person-centred communication serves as a means through which clinicians can facilitate person-centred care, enabling clinicians to gain a holistic view of a patient^20^ by sharing information and decisions, providing compassionate and empowering care, and being sensitive to needs.^20^ For instance, conveying information in line with people's information needs is demonstrated to positively influence patient outcomes, such as higher patient satisfaction and overall wellbeing.^21–23^ Attending to emotional needs is shown to be beneficial in terms of information recall and satisfaction with the clinician and provided information among people with MCI.^24^ Person-centred communication should also include communication with relatives or other individuals that accompany the person to the memory clinic, hereafter referred to as ‘care partners’. Previous studies suggest that there is still ample room for improvement of person-centred communication in clinical practice.^19,^^25–28^ For example, people with cognitive complaints want to take an active role in decision-making, yet decisions on diagnostic testing are often already made prior to the consultation by the clinician.^27^ In addition, people often feel insufficiently encouraged by clinicians to participate actively in conversations in memory clinics.^19,25,26^ People visiting the memory clinic have a need to be informed and to feel known and understood as a person by their clinician,^29^ yet people's motivation for visiting the memory clinic remains unexplored in half of the consultations.^19,28^ Ideally, these needs should be addressed in memory clinic consultations to enable person-centred communication and care. However, few studies have asked people with cognitive complaints how current communication in the memory clinic could be improved in ways that effectively address their needs and preferences for receiving information and to be seen and understood as a person, let alone involve them actively in co-designing solutions.

Co-design is a well-established approach for designing solutions together with people affected, for instance by a (chronic) disease, and other relevant stakeholders, such as designers, researchers, and developers.^30–32^ Involving people affected and other relevant stakeholders in the design process is considered beneficial, since it may lead to better ideas with high originality and user value, a better fit between people's needs and implementation in a real-world setting, empowerment of those involved, and improved User eXperience (UX) and user satisfaction in the long run.^33–35^ Additionally, co-design may lower development costs, since one is likely to immediately target the right problem, in the right way, for the right people.^30,31,36,37^ Nevertheless, few studies involve people with cognitive complaints, including those with (symptoms of) dementia, in the design process from start to finish.^36^

The aim of this study is threefold and is informed by a human-centred design approach.^38^ Firstly, we aim to identify the specific aspects of communication in the memory clinic that require resolving, in collaboration with people with cognitive complaints, and to gain preliminary insights into desired features of solutions that could help to resolve the identified issue(s). Secondly, we aim to confirm the identified problem among a new, independent group of people and design solutions in collaboration with people with cognitive complaints, care partners, memory clinic clinicians, and other relevant experts. Thirdly, we aim to assess the usability and UX of the proposed solutions.

## Methods

### Study design

For this explorative mixed-methods study, we used a development process inspired by the Double Diamond model, which is a flexible framework providing an iterative structure to designing solutions that is commonly used within human-centred design.^39^ An overview of the development process is depicted in Table 1. During the entire process, advice was provided by a sounding board, comprising professionals with expertise in dementia, medical psychology, medical communication, human-centred design, and health innovation & implementation. We also had contact with two critical friends, who are experts in participatory and inclusive research. A positionality and reflexivity statement can be found in the supplements (see Supplement 1). The Medical Ethics Committee of the Amsterdam UMC, location AMC, approved the study (METC number W22_377 # 22.449). All participants provided informed consent prior to participation. For reporting, we used the COnsolidated criteria for REporting Qualitative research (COREQ) guidelines (see Supplement 3).^40,41^

**Table 1.** Design and development process inspired by the Double Diamond model.^39^

Phase	Aim:	How	Mixed-Methods approach	Double Diamond steps
Phase I: problem statement	To identify which aspect of person-clinician communication in the memory clinic is most relevant to improve according to people with cognitive complaints, resulting in a problem statement, and to gain preliminary insights into desired features of solutions to be designed.	Co-research	n/a	Discover and Define
Phase II: problem statement triangulation and develop solutions	To verify findings from phase I and to co-design prototypes of solutions with people with cognitive complaints, care partners, clinicians, and other relevant experts.	Co-design; Lo-fi prototyping; Triangulation questionnaire; Triangulation patient-public involvement (PPI) session	Co-design sessions confirmed the main conclusion from phase I and further elaborated upon the problem statement and potential solutions. The triangulation questionnaire and PPI session were used to confirm and elaborate upon findings from the co-design session.	Define and Develop
Phase III: usability & UX testing	To examine the usability and User eXperience (UX) of the digital and analogue prototype among people with cognitive complaints, care partners, and clinicians.	Concurrent think-aloud tests followed by a mini UX interview; UX questionnaire; UX focus groups	Usability and UX tests investigated the usability and UX of the two prototypes developed in phase II. Usability testing focused on encountered usability problems, whereas the UX testing also touched upon broader topics.	Develop and Deliver

## Phase I: Problem statement

Phase I aimed to identify which aspect of person-clinician communication in the memory clinic is most relevant to improve, resulting in a problem statement, and to gain preliminary insights into desired features of solutions to be designed.

### Recruitment

People with cognitive complaints were recruited to collaborate as co-researchers. Co-researchers are people with lived experience, such as people with dementia, who collaborate in the research project as partners instead of participants, for instance, via being involved in shaping, drafting, and interpreting the findings to make the problem statement collaboratively.^42–44^ Co-researchers and facilitators worked together on a partnership level according to the ladder of participation,^45^ in which power and decision making is shared and co-researchers gain autonomy, instead of merely being listened to or participate in a study. Inclusion criteria to be a co-researcher in the current study comprised having cognitive complaints and having visited a memory clinic, whereas exclusion criteria comprised not being able to express oneself in a group conversation and/or not speaking Dutch. We conducted community recruitment, including community centres, public libraries, local organizations, supermarkets, and local health facilities across different neighbourhoods of the city of Amsterdam to maximize the chance on diversity among participants (e.g., in terms of memory clinic location, type of cognitive complaints, disease stage, and sociodemographic factors). Recruitment occurred in-person and, where possible, a short introduction presentation was given with an example exercise to help people understand what co-research entailed and induce feelings of self-efficacy among potential co-researchers.

### Procedures

Three facilitators (DV, HT, TR) facilitated eight sessions from March to June 2023 in a local community centre in Amsterdam. Each session lasted approximately one hour and started with an informal chat, followed by a discussion related to the research question (see Supplement 4 for detailed information on these sessions).

### Data collection

**Figure 1.**
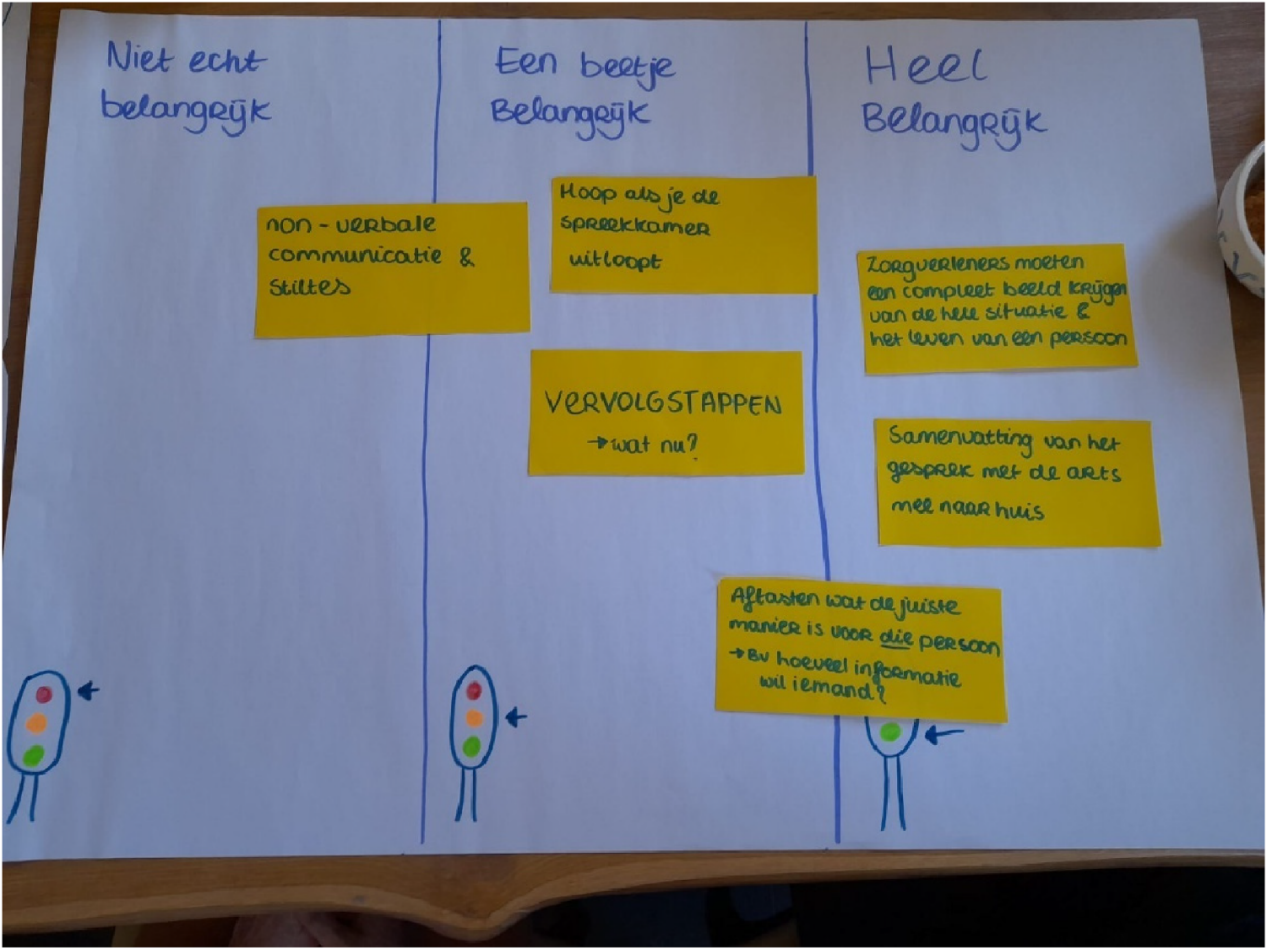
Exemplary output of the exercise during which co-researchers had to rank key insights, with ranking categories ‘not important’, ‘a bit important’, and ‘very important’.

Field notes and output of co-research exercises and/or group conversations were collected. We conducted exercises to explore and identify which aspect of communication in the memory clinic is most relevant to improve, such as via photo elicitation or stakeholder mapping (see Supplements 1 and 4 for more information on the exercises). About half-way in the fourth session, co-researchers and facilitators collaboratively mapped and categorized all insights so far, resulting in six key insights. Co-researchers ranked these six key insights from one being most important to six being least important to improve (see Figure 1 for exemplary output).

### Data analysis

When time allowed, analysis on thematic content of output of the co-research exercises and/or group conversations was done collaboratively during each session via inductive coding to identify patterns of meaning (themes) in the defined codes.^46^ In case of too little time, the facilitators (DV, HT, TR) analysed the data afterwards and discussed in a consensus meeting to inform the preparation of the next session and the creation of a paper summary of the session for co-researchers to take home. During the next session, the consensus result was presented to the co-researchers and changes to the themes and results were made when necessary.

## Phase II: Problem statement triangulation and developing solutions

The aim of phase II was to verify findings from phase I and to co-design prototypes of solutions with people with cognitive complaints, care partners, clinicians, and other relevant experts.

### Recruitment

For phase II, we recruited a more heterogeneous group of people as co-designers, involving people with SCD and MCI, care partners, and clinicians. Co-designers and facilitators worked together on a partnership level according to the ladder of participation^45^, in which power and decision making is shared and co-designers gain autonomy, instead of merely being listened to or participate in a study. A study nurse at a local academic memory clinic recruited people with cognitive complaints and care partners using a preselected list based on inclusion criteria, comprising people with SCD or MCI who visited the memory clinic in the past year, care partners of people with SCD or MCI who visited the memory clinic in the past year, and clinicians working in a memory clinic setting. Other inclusion criteria comprised being able to collaborate with others and being able to express one's opinion. Clinicians were recruited via the local memory clinic. Recruitment consisted of an email (clinicians) or a phone call (people with cognitive complaints and care partners) explaining the project and its purpose, resulting in ten co-designers. As for the triangulation questionnaire, people with cognitive complaints and clinicians were recruited via the local memory clinic and care partners via Alzheimer Nederland (Dutch Alzheimer's Association). Also, an international patient-public involvement (PPI) focus group was held in collaboration with Alzheimer Europe for triangulation purposes with 18 members of the advisory board of the European consortium projects LETHE (www.lethe-project.eu), Multi-MeMo (www.multi-memo.eu), and EU-Fingers (www.eufingers.com), comprising people with SCD, MCI, and dementia, and care partners.

### Procedures and data collection

In total, we had six co-design sessions running from November 2023 until April 2024 in the Amsterdam UMC hospital in Amsterdam. Each session lasted 1.5 hours and started with informal chatting and a creative warming-up exercise to facilitate and induce group building, creative thinking, and collaborative creation (see Supplements 1 and 5 for detailed information on the exercises and sessions). Hereafter, co-design exercises were conducted, followed by a plenary discussion. Examples comprise experience journey mapping or low-fidelity (lo-fi) prototyping.

The triangulation questionnaire was sent out in March 2024 to a broader population of people with cognitive complaints, care partners, and memory clinic clinicians to verify findings from the first three co-design sessions, during which we further defined the problem statement and explored initial ideas and/or features for possible solutions (see Supplements 6, 7, and 8 for questions). The international PPI session focused on creating a personal list of topics that people found important to discuss with their memory clinic clinician, in line with the potential solution suggested in the co-design sessions so far.

Based on the lo-fi prototypes suggested by co-designers, input from the triangulation questionnaire, and triangulation PPI focus group session, and in close collaboration with design agency Kaliber Interactive,^47^ both a digital (website) and analogue (booklet) prototype of the solution called ‘Helder in Gesprek’ (‘Clear in Conversation’) were developed in Spring 2024. The content of the prototypes will be elaborated upon in the result section.

### Data collection

For the co-design sessions, field notes, and output of exercises and/or group conversations were used as data source. Participants received a PowerPoint summary via email after each session for validation and results were discussed again at the beginning of the next session.

The triangulation questionnaire contained three questions on demographics, twelve questions on the verification of the outcomes of phase I and phase II, and seven questions on design preferences regarding the prototype (see Supplements 6, 7, and 8 for questionnaires).

Data collection throughout the PPI focus group session comprised enriching the list of topics that people would like to discuss with their clinician. PPI members could write, draw, build, and create their answers using a variety of materials (e.g., post its, images, Lego, crafting materials).

### Data analysis

When time allowed, analysis on thematic content of the output of co-design exercises and/or group conversations was done collaboratively during each co-design session using inductive coding to identify patterns of meaning (themes) in the defined codes.^46^ In case of too little time, the facilitators (KK, HT, TR) analysed the data afterwards and discussed the results in a consensus meeting, to inform the preparation of the next session and the creation of the PowerPoint summary. During the next session, the consensus result was discussed with the co-designers and results or codes were adjusted when needed.

The quantitative data from the triangulation questionnaire was analysed via descriptive statistics in SPSS Version 28.^48^ Open questions and output of the triangulation PPI session were analysed via thematic content-analysis by two researchers (KK, TR) and discussed together to reach consensus.

## Phase III: Usability and UX testing

Phase III aimed to examine the usability and User eXperience (UX) of the digital and analogue prototype among people with cognitive complaints, care partners, and clinicians using concurrent think-aloud tests followed by brief interviews, questionnaires, and focus groups.

### Usability and UX testing using a concurrent think-aloud method followed by a small UX-interview and questionnaire

Usability and UX testing, using a concurrent think-aloud method followed by a small UX interview and questionnaire,^49,50^ took place in two local memory clinics in Amsterdam and Den Bosch from September until October 2024. Tests were performed with hard- and software facilities, such as a laptop, provided by the eHealth Living & Learning Lab at the Amsterdam UMC (eHealth living & Learning Lab Amsterdam UMC).

#### Recruitment

Participants comprised people with cognitive complaints who had visited a memory clinic in the last year, and clinicians working in a memory clinic. In order to participate, a person had to be able to formulate his/her opinion. Clinicians pre-selected potential eligible participants, which were hereafter contacted by the researchers to provide information and ask for their participation. We recruited 6–8 participants per group to test either the analogue or digital prototype, since 6–8 participants are often sufficient for data saturation.^51^ Due to resource constraints, we could not conduct separate usability and UX tests for each prototype across groups with different cognitive abilities (e.g., dementia, MCI, or SCD). Participants with cognitive complaints were randomly assigned to test either the digital or analogue version, while clinicians tested both versions. Participants with cognitive complaints tested parts of the tool meant for patients visiting the memory clinic, whereas clinicians tested parts of the tool meant for clinicians.

#### Study procedures and ecological setting

To guarantee confidentiality, the provision of a comfortable environment, and the minimization of distraction, only individual participants and facilitators (and when applicable care partners) were present during the usability tests. During concurrent think-aloud testing, participants were asked to speak their thoughts out loud to get more insight into the thought processes and problems they experience while using the prototype, which they got to practice prior to the test.^49,50^

The think-aloud sessions were recorded on audio and video, and supplemented with field notes, making it possible to record users’ verbalizations as well as how their hand interacted with the prototype. The recording material consisted of two video recorders, a smartphone, two audio recorders, and one secured laptop containing Viso software with additional security by hardware keys.^52^

Participants sat in front of the digital or analogue prototype, the facilitator sat next to the participant, and the person supporting the lab technology sat opposite to the participant behind the laptop.

After introductions and an explanation of the set-up and purpose of the study, participants were asked to complete the first part of a questionnaire containing demographic information (e.g., age and gender; see Supplements 11 and 12 for the questionnaire). Hereafter, participants had to complete the tasks (see Table 2). Next, participants completed the second part of the questionnaire assessing usability, and broader user experience, containing items of the Technology Acceptance Model (TAM),^53^ Net Promotor Score (NPS),^54^ use of digital devices at home (what and how often), health literacy (FCCHL; scale 1),^55^ and digital health literacy (DHLI for patients and care partners; self-perceived digital literacy for clinicians).^56^ Finally, a short interview was conducted to capture UX and additional feedback regarding the user experience of the prototype (e.g., including PrEmo, a validated, cross-cultural method to measure people's emotions towards a design^57^; see Table 2).

**Table 2.** Tasks and interview questions for usability testing.

Analogue prototype	
Practice task	
	In the booklet, you can answer statements about yourself with yes or no. Can you please find the first statement?
Tasks	
	1. Flip through the booklet. Can you find the four themes of the statements for me?
	2. There is a page in the booklet with instructions on how to use the booklet. Can you find this page for me?
	3. You may now go back to the first proposition. You may go through the booklet again until you find a statement that applies to you. Can you then complete the questions on this statement for me (2x)?
	4. In the booklet, you may also think of and add your own statement. Can you find for me the place in the booklet where you can add your own statement?
	5. You now have two statements that you would like to discuss with your healthcare provider. Can you put them in the right place in the booklet for me?
	6. You can make notes in the booklet. Can you find the place in the booklet where you can make notes for me?
**Digital prototype**	
Practice tasks	
	On this phone, can you please look up the weather forecast for upcoming Saturday?
	On the website, you can find contact information of the Amsterdam UMC in case you have any questions. Could you please look up the contact information?
Tasks	
	1. On the website you can answer statements about yourself with yes or no. Can you answer these statements for me?
	2. If someone wants to change some of the statements, you can. Can you change for me an answer to the statements?
	3. In the next step, you can give an explanation the statements. Can you give an explanation of four statements for me?
	4. In the next step, you can summarize the topics you would like to discuss with your healthcare provider. Can you make a summary for me?
	5. In the next step, you can make a summary and download it or send it to your memory clinic. Can you send your summary to your own e-mail address for me?
**Post-usability test interview guide**	
Questions	
	1. What did you think about your participation in this study?
	2. From these puppets [PrEmo], could you please choose the one that best reflects how you feel about the tool? And why?
	3. How did you feel about filling in statements?

#### Data analysis

The audio of these sessions was transcribed verbatim. Transcripts were enriched with observations from the video recordings. The verbatim transcripts were analysed alongside usability metrics (i.e., errors), field notes, and video annotations in an Excel file. Usability problems were coded using open coding (coding the smallest piece of text describing a usability problem in a line-by-line manner) and axial coding (organizing these smallest pieces of meaning into bottom-up categories), followed by deductive coding based on the DEMIGNED principles by LH and TR. LH and TR discussed the results until consensus was reached. In the case of doubts or discrepancies, TE was consulted. The DEMIGNED principles describe actionable design considerations that can be applied in the development of health technologies for people with dementia, for instance, including principles on navigation and positive feedback.^58^ The overarching categories of the DEMIGNED principles comprise barriers related to cognition (problems with cognitive abilities may result in difficulties with understanding, remembering, or interacting with digital systems; e.g., easy navigation to functions and content), perception (problems with the ability to visually and sensorially identifying interface components may result in difficulties with effective interaction and accessibility; e.g., appropriate system feedback), frame of mind (design elements that consider and support the emotional and psychological well-being of users, ensuring the system is both functional and affirming; e.g., positive feedback for correct action completion), and speech and language (communication difficulties highlight the need for information that is clear, accessible, and supportive; e.g., understandable words and sentences that feel comfortable)’.^59,60^ Questionnaire responses were analysed in SPSS (version 28)^48^ using descriptive statistics.

### User testing: UX focus group and UX questionnaire

UX focus groups took place in community centres and organizations in Amsterdam, Eindhoven, Amersfoort, or via online Microsoft Teams meetings from August until November 2024. We conducted focus groups to gain insight in UX and to substantiate the findings from the usability testing, investigating whether the proposed tool matched users’ needs, preferences, and memory clinic flow among a broader sample.

#### Recruitment

Recruitment was done at memory clinics, community organizations (e.g., local community centres) and professional organizations (e.g., the Dutch network of memory clinic professionals and Alzheimer Nederland (Dutch Alzheimer's association)) and consisted of a phone call or e-mail explaining the project and its purpose (by KK, TR). In order to participate, a person had to be able to express their opinion and must have visited the memory clinic in the past two years or work as a clinician in a memory clinic. When needed, we performed purposive sampling and conducted one-on-one semi-structured interviews addressing the exact same topics if a focus group was not feasible. Participants consisted of people with cognitive complaints and care partners who had visited the memory clinic last year, and memory clinic clinicians. We aimed to recruit 15 per focus group, as 6–12 participants is often enough for data saturation and aiming for 15 participants leaves room for drop-outs.^61^

#### Data collection

Focus groups started with collaboratively going through the analogue and digital version of the tool. Hereafter, questions were asked on initial impressions (e.g., using PrEmo,^57^ TAM,^53^ NPS^54^), suggestions for improvement, and preference for the digital or analogue version of the tool (see Supplement 13 for topic guide). Next, participants completed the same questionnaire as described above at the usability testing section (see Supplements 9 and 10 for questionnaire).

#### Data analysis

The sessions were audio-recorded, transcribed verbatim, and analysed using MAXQDA.^62^ Two researchers performed bottom-up coding and thematic content analysis for the concurrent think-aloud tests (LH, TR) and the brief interviews and focus groups (JH, TR). Results were discussed until consensus was reached and TE was consulted in case of any doubts or discrepancies. Questionnaire responses were analysed in SPSS (Version 28),^48^ using descriptive statistics (by TR).

## Results

In total, 108 persons were involved in the project as a co-researcher, co-designer, or participant (phase I: n = 4; phase II: n = 51; phase III: n = 51; see supplement 2 for a visual flow chart). Below, detailed results are provided per phase. For reporting, we used the COnsolidated criteria for REporting Qualitative research (COREQ) guidelines (see supplement 3).

## Phase I: Problem statement

During phase I, we started with four co-researchers with dementia (female: 50%). One co-researcher dropped out half-way due to personal circumstances. Table 3 provides an overview of insights ranked from most important (number 1) to least important (number 6) to improve according to the co-researchers.

**Table 3.** Key insights ranked from most important to least important to improve according to co-researchers when it comes to person-clinician communication in the memory clinic.

Clinicians need to get a complete picture of a person's life and circumstances A summary of the consultation to take home Tailored communication by the clinician → e.g., how much information does someone want? Discuss follow-up steps The feeling of hope as you walk out of the consultation room Non-verbal communication and silence provided by the clinician

Key insights 1 and 2 were considered important by all co-researchers. Since the facilitators (DV, HT, TR), were aware that some Dutch memory clinics already provide summaries of the consultation for patients to take home, they focused on key insight number 1 from then on. Co-researchers desired clinicians to get a comprehensive understanding of someone's life and circumstances. They would, for instance, like their clinician to get insight into relevant aspects of their life history and ways of coping. According to the co-researchers, the sooner a clinician is able to get a comprehensive understanding of who they are and what is important to them, the better. They also highlighted that it would be valuable if clinicians would ask input from the care partner, who might be able to provide additional pieces of information about the person with cognitive complaints. However, co-researchers also stressed that getting a comprehensive understanding of someone's life and circumstances only needs to pertain to aspects with some degree of relevance for the complaints someone is experiencing and the reasons they have for visiting the memory clinic.

When talking about a potential tool that could support person-clinician communication, the following features were deemed desirable by the co-researchers: concise, possible to use with others (e.g., with care partner), facilitating repeated use, linguistic rather than purely visual (but this may not be suitable and accessible for everyone), possible to use at home (preferably on the go as well), more serious than fun, 2D or on paper, ‘old fashioned’ (as this is ordinary and familiar to the target audience), comprise information on someone's past and present, and is generic (with a few standard steps).

## Phase II – Problem statement triangulation and developing solutions

In total, eight co-designers were included, consisting of people with subjective cognitive complaints (n = 3; mean age = 68 years; female: 1/3; educational attainment low (2/3) or medium (1/3)), care partners (n = 2; mean age=67 years; female: 1/2; educational attainment score high (1/2) or medium (1/2)), and clinicians (n = 3; mean age=29; female: 3/3; educational attainment: high (3/3); profession: specialized nurse (1/3), care counsellor (1/3), and medical doctor (1/3)). When asked what co-designers would find important that clinicians know about their patients, many aspects were mentioned (see Table 4 for examples).

**Table 4.** Overview of exemplary topics in random order that co-designers, participants in the triangulation questionnaire, and triangulation PPI session deemed important for clinicians to know about their patients.

Whether I want to have an active lifestyle The emotional impact my symptoms have on me My image of the future My frustrations (e.g., what is no longer possible) My way of coping/problem-solving regarding my cognitive complaints How I perceive life Whether I want to prepare myself for the future or not Whether I easily ask for support/help What kind of hobbies I have Whether I work or not What my home situation is like What my responsibilities in life are Whether I can get support from my partner, family, friends, and/or other loved ones Whether I have connections with others Whether I seek contact with others or rather withdraw

These topics partially overlapped with topics identified by means of the triangulation questionnaire (total n = 25), which was completed by people with cognitive complaints (n = 7; mean age=66 years; female: 4/7; educational attainment low (1/7), medium (2/7), or high (4/7)), care partners (n = 6; mean age=61 years; female: 6/6; educational attainment low (1/6), medium (3/6), or high (2/6)), and clinicians (n = 12; female: 10/12), and in the PPI focus group session with the advisory board members (n = 18) of the LETHE, Multi-MeMo, and EU-Fingers projects (see Supplement 9).

Although the majority of people with cognitive complaints and care partners in the triangulation questionnaire reported to experience little problems in sharing personal information with their clinician, they did find it important that clinicians obtain a comprehensive understanding of their life and circumstances (57% people with cognitive complaints; 40% care partners; see Supplement 9). Creating lists containing one's questions prior to the consultation was mentioned as a facilitating strategy for sharing personal information with the clinician. Fear, nerves, and experiencing the clinician to be time-pressured or having a lack of personal attention withheld people with cognitive complaints and care partners from raising topics they would like to discuss. Clinicians had a generally positive attitude towards developing a solution that facilitates people with cognitive complaints and care partners in sharing personal information, as long as the solution would be efficient and usable, and not overlap with existing questionnaires. People with cognitive complaints, care partners, and clinicians had no clear preference for either a digital or analogue solution (see Supplement 9).

Co-designers indicated that people should feel reassured, listened to, understood, at ease, and feel regarded as a person after using the solution. When discussing with co-designers about where in the patient journey people could be supported with a solution, the consultation with a clinician itself was mentioned. Clinicians should explicitly mention that there is time to discuss personal information that someone wants to share and that they will listen. Co-designers strongly recommended that it should also be possible to use the solution prior to the consultation, so that one can prepare. This preparation may reduce anxiety about the possibility of forgetting what people want to discuss with the clinician. Co-designers indicated that it should be possible to use the solution together with someone else, such as a care partner. Some co-designers also thought that the solution could be used after the consultation, to reflect upon what has been said and help to identify any remaining needs. Lo-fi prototyping of the solution resulted in a list of exemplary types and desired features of the prototype (see Table 5 for example; see Supplement 5 for a description of the exercise). Co-designers are open to using both an analogue and digital version of the solution. Ideally, the digital version should be compatible with a tablet or mobile phone, followed by a computer or laptop, or an e-reader.

**Table 5.** Example of a lo-fi prototype and its features by co-designers.

Example lo-fi prototype	Example features
Pictograms/vision board 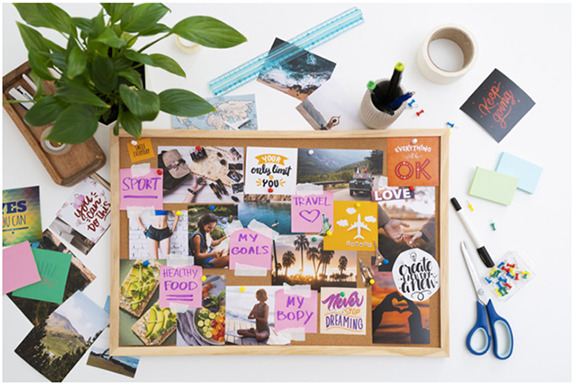	game-like elements may contain personal information (e.g., pictures, documents, or letters) physical tool or on a tablet or smartphone to be used together with a care partner or clinician visual information something to be able to point at if talking is difficult

Based on all insights from phase II, a digital and analogue prototype was made in collaboration with design agency Kaliber Interactive 47 (see Box 1).

Box 1. The digital and analogue prototype of ‘Helder in Gesprek’

**Figure 2.**
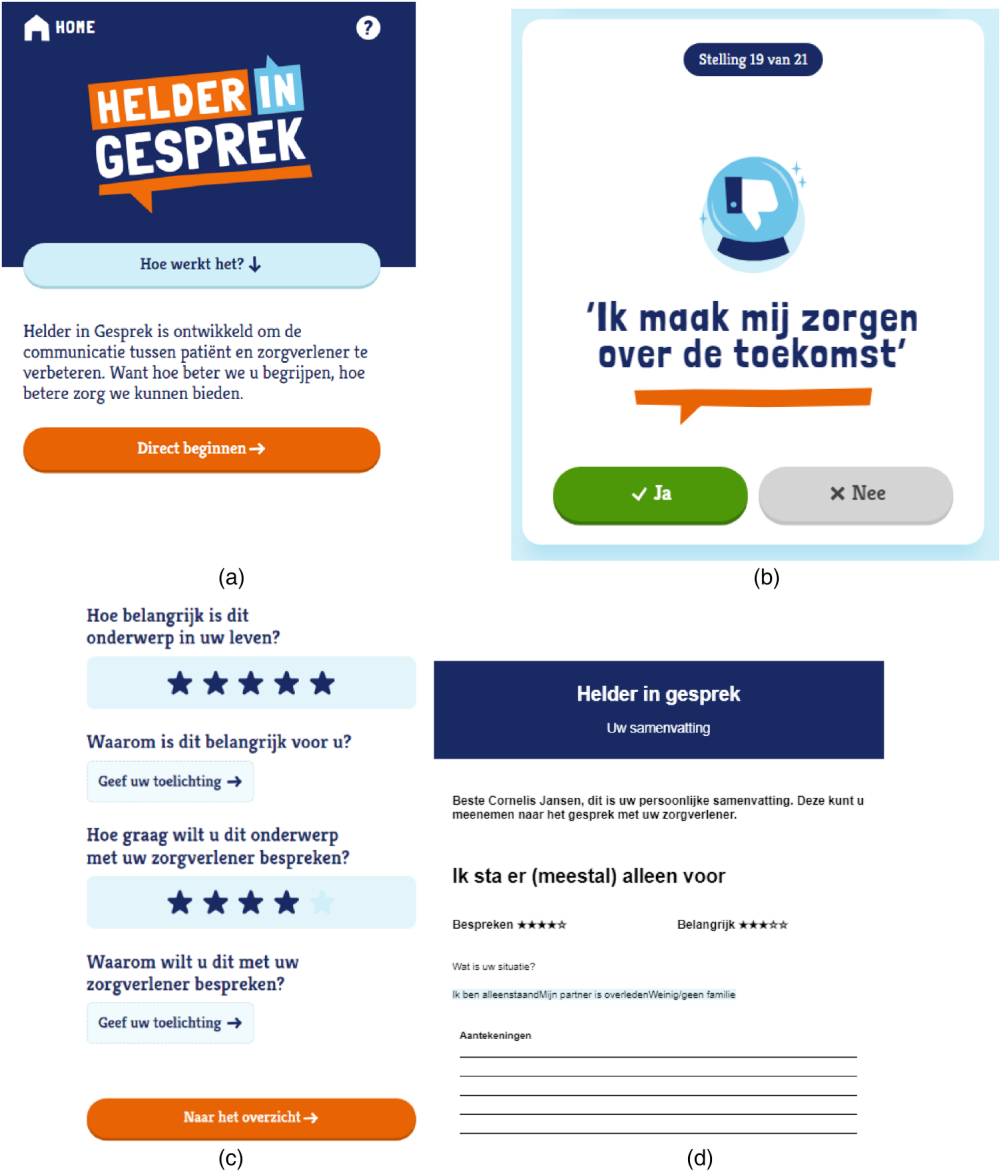
(a) home page of helder in gesprek [digital prototype]. (b) example of a statement; (c) example of follow-up questions per statement; (d) example of a summary page.

Two prototypes of the tool ‘Helder in Gesprek’ were developed: an analogue prototype in the form of a booklet and a digital prototype in the form of a web-based tool (literal translation: ‘Clear in Conversation’; ^©^2025-ABOARDxKaliber. All rights reserved.) (see Figure 2(a); see Supplement 10 for how phase I and phase II informed prototype design). We developed the tool in collaboration with the formal design company Kaliber. The aim of ‘Helder in Gesprek’ is to help people think about topics that are important to them at this moment in time and which of these topics they would like to discuss with their memory clinic clinician. For instance, topics related to who they are as a person, their circumstances, and needs. People with cognitive complaints can use the tool at home by themselves or together with a care partner. Clinicians receive a summary of the result and can therewith quickly identify what is most important to discuss with a specific person. ‘Helder in Gesprek’ thus helps to facilitate people visiting the memory clinic in discussing topics relevant to them so they feel more seen, heard and understood.

Both prototypes start with a general introduction followed by 21 statements. People can answer the statements with either ‘yes’ or ‘no’ (see Figure 2(b)). If someone chooses ‘yes’, they are prompted with follow-up questions to indicate 1) why this topic is important to them and 2) the relevance of discussing this topic with their memory clinic clinician (see Figure 2(c)). At the end, people can make an overview of the three most relevant topics. When using the digital prototype, suggestions are automatically generated for the three topics most important to discuss with their memory clinic clinician (see Figure 2(d)).

## Phase III – Usability and UX testing

In total, 17 people with cognitive complaints tested the digital or analogue prototype using a concurrent think-aloud test followed by a UX questionnaire. Participants were on average 70 years old, Dutch, and had a smartphone (see Table 6a). Their technology acceptance (TAM) score for ‘Helder in Gesprek’ was relatively low suggesting that they experienced a low level of acceptance or willingness to use ‘Helder in Gesprek’ (see Table 6a). In the UX focus group sessions, 13 people with cognitive complaints participated mainly consisting of Dutch women in their seventies with a dementia diagnosis (see Table 6b). Care partners in UX sessions (n = 4) were on average 48 years old, Dutch, had a smartphone, and had a relatively low technology acceptance (TAM) score (see Table 6c). Clinicians (n = 17) were on average 40 years old, Dutch, and had a relatively low technology acceptance (TAM) score (see Table 6d). Co-researchers and co-designers from phase I and phase II continued to provide input via attending the UX sessions.

*Data in the pictures is based on an imaginary use case.

**Table 6a.** Study sample usability - participants with cognitive complaints (total n = 17; digital tool n = 9; analogue tool n = 8).

Sociodemographics	
Age, mean	69.3 ± 5.3
Gender	58.8% female (n = 10)
Educational attainment	Low: 5.9% (n = 1) Medium: 29.5% (n = 5) High: 64.7% (n = 11)
Diagnosis	SCD: 17.6% (n = 3) MCI: 23.5% (n = 4) Dementia: 11.8% (n = 2) No diagnosis: 47.1% (n = 8)
Language spoken at home	Dutch: 82.4% (n = 14) Dutch and English: 17.6% (n = 3)
Country of birth	Netherlands: 94.1% (n = 16) Sweden: 5.9% (n = 1)
Use of digital tools	Smartphone: 100% (n = 17) (52.9% Android phone (n = 9); 47.1% Apple phone (n = 8)) Laptop or desktop computer: 88.2% (n = 15) Tablet: 88.2% (n = 15)
Total health literacy (FCCHL)	2.2 ± 0.7 (min=1; max=3.5)
Total digital health literacy (DHLI)	64.1 ± 12.7 (min=33; max=83)
*Outcomes*	
Perceived usefulness (subscale TAM)	1.3 ± 0.5 (min=1; max=3)
Perceived ease of use (subscale TAM)	1.5 ± 0.8 (min=1; max=4)
Attitude to use (subscale TAM)	1.4 ± 0.8 (min=1; max=4)
Behavioural intention (subscale TAM)	1.6 ± 1.1 (min=1; max=5)
Perceived satisfaction (NPS)	7.9 ± 2 (min=3; max=10)

**Table 6b.** Study sample UX – participants with cognitive complaints (n = 13).*

Sociodemographics	
Age, mean	76.5 ± 10.2 N = 9 missing
Gender	Female: 69.2% (n = 9)
Educational attainment	High: 23.1% (n = 3) Missing: n = 10
Diagnosis	Dementia: 92.3% (n = 12) No dementia: 7.7% (n = 1)
Language spoken at home	Dutch: 100%
Country of birth	The Netherlands: 84.6% (n = 11) Malaysia: 7.7% (n = 1) New Guinea: 7.7% (n = 1)

*Asking for health literacy, digital literacy, use of digital tools, and TAM subscales in the limited time available was not deemed feasible.

**Table 6c.** Study sample UX – care partners (n = 4).

Sociodemographics	
Age, mean	47.5 ± 22.8
Gender	50% female (n = 2)
Educational attainment	Medium: 25% (n = 1) High: 75% (n = 3)
Language spoken at home	Dutch: 100% (n = 4)
Country of birth	Dutch: 100% (n = 4)
Use of digital tools	Smartphone: 100% (25% (n = 1) Android; 75% (n = 3) Apple) Laptop or desktop computer: 75% (n = 3) Tablet: 50% (n = 2)
Total health literacy (FCCHL)	2 ± 0.4 (min=1.5; max=2.5)
Total digital health literacy (DHLI)	73.8 ± 3.3 (min=70; max=77)
*Outcomes*	
Perceived usefulness (subscale TAM)	1.7 ± 0.7 (min=1; max=2.75)
Perceived ease of use (subscale TAM)	1.3 ± 0.5 (min=1; max=2)
Attitude to use (subscale TAM)	1.8 ± 0.6 (min=1; max=2.5)
Behavioural intention (subscale TAM)	2.3 ± 1.9 (min=1; max=5)
Perceived satisfaction (NPS)	8.3 ± 1.5 (min=7; max=10)

**Table 6d.** Study sample UX – clinicians (n = 17).*

Sociodemographics	
Age, mean	41.1 ± 12.5
Gender	64.7% female (n = 11)
Educational attainment	High: 100% (n = 17)
Profession at the memory clinic	Neurologist: 5.9% (n = 1) ANIOS Neurology: 5.9%*** (n = 1) Geriatrician: 11.8% (n = 2) Medical doctor (not specialized; unknown department): 5.9% (n = 1) Neuropsychologist: 47.1% (n = 8) Specialized nurse: 5.9% (n = 1) Nurse: 5.9% (n = 1) Case manager: 5.9% (n = 1) Internal medicine geriatrician: 5.9% (n = 1) Spiritual counsellor: 5.9% (n = 1) Clinical & neuropsychologist: 5.9% (n = 1)
Years of experience at the memory clinic	0–10 years: 52.9% (n = 9) 11–20 years: 29.4% (n = 5) 21–30 years: 5.9% (n = 1) 31–40 years: 5.9% (n = 1) Other: 5.9% (n = 1)
Language spoken at home	Dutch: 88.3% (n = 15) Dutch and English: 11.8% (n = 2)
Country of birth	Netherlands: 100% (n = 17)
Use of digital tools	Smartphone: 100% (41.2% (n = 7) Android; 58.8% (n = 10) Apple) Laptop or desktop computer: 88.2% (n = 15) Tablet: 58.8% (n = 10)
Self-perceived digital skills	7.5 ± 1.3 (min=5; max=9)
*Outcomes*	
Perceived usefulness (subscale TAM)	2.2 ± 1.1 (min=1; max=4.3)
Perceived ease of use (subscale TAM)	1.9 ± 0.8 (min=1; max=3.8)
Attitude to use (subscale TAM)	2.1 ± 1.1 (min=1; max=4.5)
Behavioural intention (subscale TAM)	2.6 ± 0.9 (min=1; max=4)
Perceived satisfaction (NPS): Patient	6.9 ± 1.4 (min=3; max=8)
Perceived satisfaction (NPS): Colleague	6.8 ± 1.5 (min=3; max=9)

*The usability studies with memory clinic clinicians (n = 8) are not taken into account in this study, however, these participants also filled in the questionnaire that was used in the UX study, resulting in a total of n = 17 questionnaires by memory clinic clinicians that are taken into account for this study *** ANIOS = doctor not in specialist training.

Usability tests on the digital prototype showed a total of 286 usability problem encounters, which were thematically categorized using the DEMIGNED principles into 43 unique usability problems (see Supplement 14). Common identified usability problem encounters comprised navigation (52/286; 18.2%), screen sensitivity (41/286; 14.3%), distinguishable elements (40/286, 14%), system feedback (29/286, 10.1%), and understandability (27/286, 9.4%) (see Supplement Table 4). For instance, participants did not find navigation to be clear or intuitive. Participants were sometimes unable to locate the required page, which caused confusion and frustration. Usability tests on the analogue prototype showed a total of 101 usability problems encounters, which could be thematically categorized in 31 unique usability problems (see Supplement 15). Common identified usability issues were related to distinguishable elements (17/101, 16.8%), instructions (26/101, 25.7%), understandability (12/101, 11.9%), and booklet anatomy (16/101, 15.8%) (see Supplement Table 5). For instance, the results of the analogue prototype indicate that the most common issues encountered are related to the visibility of the star rating element, the way statements and questions are interpreted, and the fact that the question ‘How important is this for you?’ has both a free text field and a star rating bar, which may confuse the user.

Post-usability interviews and UX focus groups overall indicated that most people with cognitive complaints, care partners, and clinicians had a positive attitude towards the general concept of ‘Helder in Gesprek’ (as indicated in the interview and reflected in their PrEmo scores; for PrEmo scores see Supplement 16), for instance, regarding its purpose, design, and the possibility to use the prototype together with others. However, several areas for improvement were also identified. For instance, regarding the usability of the prototypes, such as the understandability of texts in the tool, difficult navigation, and cognitive overload. Some participants thought that people with cognitive complaints were unable to use the prototypes by themselves due to the complexity, which was perceived as a negative aspect. Also, some participants thought that the purpose of why people could use ‘Helder in Gesprek’ did not come across clearly yet. Likewise, how to use the tool in clinical practice was also perceived as not coming across clearly in the current prototypes. Some participants also mentioned that the current version of the prototypes contained too much content to be used in clinical practice or questioned the feasibility in clinical practice due to limited consultation time. Clinicians wondered if it was possible to incorporate the digital prototype into an electronic patient record (for visual overview of all themes and exemplary quotes see Supplements 17 and 18).

## Discussion

This study aimed to (a) identify specific aspects of person-clinician communication in the memory clinic context that require resolving, (b) co-design solutions, and (c) assess the usability and User eXperience (UX) of the proposed solutions. Co-researchers emphasized the importance of clinicians gaining a holistic understanding of someone's life and circumstances, which was confirmed and elaborated upon in an additional questionnaire, international focus group session, and in the co-design trajectory. This resulted in a comprehensive list of topics that might be important for a person to address during a consultation with their memory clinic clinician, along with information on what moment in the patient journey would be best to prepare and discuss these topics. Initial lo-fi prototype directions were created by co-designers, followed by the development of a digital and analogue prototype of ‘Helder in Gesprek’ (literal translation: ‘Clear in Conversation’). These prototypes were tested on usability and User eXperience (UX), identifying areas for improvement, such as navigation, system feedback, clarity of content, and cognitive load. Throughout the entire study, the Double Diamond model was used as a framework. We experienced this framework as helpful to explain the structure of the current study to the co-researchers and co-designers, especially to those with cognitive complaints (see Supplement 1 for further reflections).

During phase I, co-researchers identified six key needs regarding communication in memory clinics, including the need to receive a summary of a consultation, the need for tailored communication, the need for information on follow-up steps, the need of feeling hope, and the need for supportive non-verbal communication and silences provided by the clinician. These key needs are in line with previous research in a dementia and memory clinic setting^29,^^63–66^ as well as in medical communication in other settings.^67–73^ Co-researchers agreed upon the most important need; they need their memory clinic clinician to gain a holistic understanding of their life and circumstances. This finding is in line with previous research among people with cognitive complaints or dementia, that also indicates that this need is not (always) sufficiently fulfilled.^7,^^17–19^^,29^ In the next phase, this need to feel known and understood as a person was confirmed by co-designers. However, although the importance of clinicians gaining such a holistic understanding was acknowledged and re-iterated by participants in the triangulation questionnaire, these participants on average did not report a need for communication support. They did mention barriers for drawing clinicians attention to their personal situation, such as the feeling of time constraints, perceived lack of personal attention by the clinician, and feelings of nervousness or anxiety. This is why we decided to still proceed with designing a solution that would help people to determine what they would like their clinician to know about them and support a conversation about these topics in memory clinic consultations.

In phase III, participants generally expressed a positive attitude towards the concept of ‘Helder in Gesprek’ during the UX tests. However, we also identified usability problems and areas for improvement, mainly regarding the design principle of ‘Cognition’, which refers to design that reduces barriers for use related to memory, attention, problem-solving, and information processing.^60^ Taking differences in users’ cognitive abilities into account is particularly important when designing for people with cognitive complaints. ^58,^^74–76^ Even though we aimed apply the design principles from the start, including those on cognition, we still found a considerable number of usability issues. This may be because we tested prototypes instead of fully developed tools. Navigation problems are an example of cognition-related usability problems, which occurred in both the analogue and digital prototype during usability testing. Several suggestions for improvement regarding navigation were mentioned during UX testing, such as adding tabs, table of contents, or a drop-down menu. People with cognitive complaints may experience problems related to spatial orientation and memory, which highlights the importance of a sequential or linear navigation, possibly complemented with multiple forms of navigational support elements, such as having clear ‘back’ and ‘next’ buttons or clear progress indicators, such as a checklist.^77^ A previous study assessing usability of a fully developed mobile website among people with cognitive complaints also showed a majority of usability problems that centred around navigation,^58^ highlighting the difficulty of designing for people with cognitive complaints and the necessity of conducting usability and UX tests with end-users.

The usability and UX tests also identified other usability issues. For instance, the colour contrast was sometimes found to be challenging in the digital prototype during usability testing. This may be due to the fact that colours were shades of blue, which can be hard to distinguish, particularly given that colour contrast sensitivity diminishes with age.^78,79^ In addition, although we were aware of the need to use clear, concrete, and concise texts when designing for people with cognitive problems based on previously published research and recommendations,^80–82^ the texts in the tools were nevertheless considered too complicated in both prototypes during both usability and UX testing.^59,60^ The texts in the tool, for example, were found to be too complex and/or redundant, for instance, including statements containing negations (e.g., the statement ‘I do not work (anymore)’). Understandability issues may also cause difficulties in recognizing certain functionalities in the protype among people with cognitive complaints, further undermining the usability. The final overarching usability problem was cognitive overload, which was found during both usability and UX testing. Participants found both prototypes overwhelming in the number of steps and amount of information provided, which frequently co-occurred with navigation problems. For instance, participants sometimes forgot which task they had to fulfil when flipping through the instruction pages or statements in the analogue prototype. Based on the feedback we received, we realized that, despite good efforts and intentions when designing, our prototypes could greatly be improved by taking into account all identified issues and recommendations that resulted from usability and UX tests. The most important recommendations that we distilled from our findings are to improve the navigation, understandability, system feedback, distinguishable elements, screen sensitivity, instructions, booklet anatomy, clarity of content, and reduce cognitive overload. These areas for improvement may also guide the development of other communication tools in a healthcare setting.

PrEmo scores (Product Emotion Measurement Instrument) indicated that most people with cognitive complaints, care partners, and clinicians had a positive attitude towards the concept of ‘Helder in Gesprek’. Yet, this positive attitude was not reflected in clinicians’ Net Promoter Scores (NPSs) indicating that clinicians are not likely to use the tool in its current form. This discrepancy may indicate a positive baseline attitude regarding the concept of ‘Helder in Gesprek’ (i.e., person-centred communication in the memory clinic), but not regarding the current prototypes, highlighting a need for further re-design. Another explanation could be that PrEmo scores were asked verbally whereas NPS scores were collected on paper and the latter method might reduce social desirability in clinicians’ answers. ^83–86^ Also, both the NPS and PrEmo in this study consist of one question, which may be insufficient to capture nuance in people's attitude towards a prototype. Future research is recommended to measure the attitude towards a tool using multiple measures with opportunities for follow-up questions to capture nuances and limit a social desirability bias. Also, the attitude of clinicians towards ‘Helder in Gesprek’ is an important factor to address in further development and testing.

This study described the development of both a digital as well as an analogue prototype. As mentioned, the analogue prototype resulted in less usability problems compared to the digital prototype. This is not surprising, since the digital prototype contained more detailed functions and complex navigation, functionalities, and top-down texts compared to the analogue protype. Moreover, the digital prototype disallowed an individual to continue when they make a mistake, which may have induced frustration. Lastly, the modality of an analogue prototype may suit people with more severe cognitive complaints better compared to the digital prototype. During both usability and UX tests, people with dementia or MCI showed and/or explicitly mentioned to have more difficulties with using the digital prototype, for instance related to cognitive overload, and the majority preferred an analogue solution. In contrast, people with SCD completed tasks in the digital prototype rather smoothly and/or explicitly mentioned a preference for a digital prototype. For future re-design, we will prioritize re-designing the analogue prototype to cater towards those who may need support the most. Another important argument for further development of the analogue prototype comprises inclusivity in relation to digital inequality. The latest Digital Economy and Society Index by the European Commission shows that 17.3% of the Dutch population does not have at least basic digital skills.^87^ When looking specifically at the field of dementia, we know from previous research that many risk factors for dementia are related to existing inequities in accessing (digital) health care, that is, occurring more often in minority groups, such as people with a lower socioeconomic position.^88,89^ From an ethical perspective, it is crucial to be able to ensure the usability and accessibility of memory clinic-related tools for all, and therefore not to go (fully) digital as of yet.

Among the strength of our current work is the co-research and co-design approach. Not only did people like to be involved throughout the entire design and development study, they also felt heard, encouraged, and valued, and they had fun during the sessions. Another strength is the triangulation of data collection methods and sources, and the involvement of 108 individual people throughout the entire study. Some limitations are also worth mentioning. First, some co-researchers and co-designers experienced difficulties in recalling previous sessions, despite efforts to support memory (e.g., via roadmaps and summaries). Similarly, although we tried to make the sessions as concrete as possible, whilst also leaving enough space for creative input, some exercises were nevertheless too abstract and cognitively demanding. Although alternative methods were employed to capture input from these exercises, this approach may have resulted in reduced depth in some responses. Second, although we worked with people with varying cognitive abilities, educational backgrounds, ages, life stages, and living in municipalities across the Netherlands, diversity could have been more optimal in terms of culture, socioeconomic backgrounds, health literacy, digital literacy, and insights from those who need to build more trust before engaging in research or design projects. We tried to maximize diversity via community recruitment. However, due to limited time for recruitment, there was little room to build rapport and a location for the sessions could not always be offered in the neighbourhood that someone was living in. Also, all recruitment material and procedures were in Dutch, which may have excluded some from becoming involved. Therefore, the findings of this study may not be generalizable to all individuals attending the memory clinic, and future research involving a more diverse sample, reflecting the previously mentioned characteristics, is warranted. Third, during usability testing, clinicians tested both the analogue and digital prototype in one session. However, we did not randomize the order in which they tested the prototypes, potentially leading to an order effect: the experience with the first, digital prototype may have shaped their expectations, performance, and verbalized thoughts during the second think-aloud test with the analogue prototype. This may have influenced the results, because participants may have been familiar with the type of tasks that they needed to perform. Also, the order may have negatively affected their attitude of the analogue prototype, since the digital prototype for clinicians appears more concise compared to the analogue prototype. Fourth, we did not use validated questionnaires limiting our internal validity. Instead, we used the validated questionnaires to inform the questions in our questionnaire, such as the functional scale of the Functional, Communicative, and Critical Health Literacy (FCCHL) scale,^90,91^ the Digital Health Literacy Instrument (DHLI),^56^ and the Technology Acceptance Model.^53^ We translated these questions to Dutch and, when necessary, made changes in the language used so that all questions were formulated at language level B1 for clarification purposes. Fifth, during usability testing, we were unable to conduct subgroup analyses on usability among people with SCD, MCI, and dementia since the number of inclusions per groups was too small to make meaningful comparisons in usability (SCD: n = 3; MCI: n = 4; dementia: n-2; no diagnosis: n = 8), as 6–8 participants per group are usually needed for data saturation during usability testing.^51^ As mentioned above, observations seem to suggest poorer usability among people with dementia compared to people with SCD or MCI, however, more research is needed to confirm any subgroups differences. Sixth, facilitators were responsible for conducting the thematic analysis and leading the consensus meetings, which may have resulted in confirmation bias. Seventh, we did not perform inter-coder reliability during thematic analysis, which limits the internal validity. Finally, usability tests with the digital protype were conducted on an iPhone, which may have led to some usability problems for people who are used to Android smartphones.

Future research should engage in re-design of the prototypes based on the feedback from the usability and UX tests and conduct subgroup analyses on usability among people with SCD, MCI, and dementia. Hereafter, a feasibility study on ‘Helder in Gesprek’ should be conducted in clinical settings, preferably using implementation frameworks, such as the Knoster framework, Kotter's 8-step model, or the Consolidated Framework for Implementation Research (CFIR).^92,93^ Such a feasibility study should also gain initial insight into whether the tool can enhance person-centred communication in the sense that people visiting the memory clinic feel more seen, heard, and understood. Besides people with cognitive complaints, care partners, and clinicians, it is important to explicitly include other key stakeholders as well in further testing and implementation activities. For instance, policy makers as they can allocate resources and backing to support implementation.

## Conclusion

This study identified a key need among people visiting memory clinics: the importance of clinicians gaining a holistic understanding of someone's life and circumstances. This is a need that also arose from previous research, and that was confirmed and elaborated upon in our co-design trajectory, and by means of two additional data collections (a questionnaire and focus group session) that we conducted for triangulation purposes. Through a co-research and co-design process, we developed both a digital and analogue prototype of the solution ‘Helder in Gesprek’. ‘Helder in Gesprek’ aims at supporting people with cognitive complaints and potentially their care partners in preparing for memory clinic consultations with the goal to engage in a meaningful conversation on the aspects of their life and circumstances that they consider important to share with their clinician. Usability and UX testing revealed several areas for improvement, such as navigation, understandability, system feedback, distinguishable elements, screen sensitivity, instructions, booklet anatomy, clarity of content, and reduce cognitive overload. These findings will inform concrete recommendations for re-design. Future research should evaluate the feasibility and implementation of ‘Helder in Gesprek’ in real-world clinical setting. The insights from this study may also guide the development of other communication tools in a healthcare setting.

## Supplemental Material

sj-docx-1-dhj-10.1177_20552076251412631 - Supplemental material for Design and development of ‘Helder in Gesprek’: A tool to support person-centred communication in memory clinics Supplemental material, sj-docx-1-dhj-10.1177_20552076251412631 for Design and development of ‘Helder in Gesprek’: A tool to support person-centred communication in memory clinics by Tanja J de Rijke, Kyra KM Kaijser, Dianne Vasseur, Hilal Tasköprü, Lotte Huisman, Aniek M van Gils, Vera Otten, Carolien Smits, Cynthia S Hofman, Minke Kooistra, Ellen MA Smets, Thomas Engelsma and Leonie NC Visser in DIGITAL HEALTH

sj-pdf-2-dhj-10.1177_20552076251412631 - Supplemental material for Design and development of ‘Helder in Gesprek’: A tool to support person-centred communication in memory clinics Supplemental material, sj-pdf-2-dhj-10.1177_20552076251412631 for Design and development of ‘Helder in Gesprek’: A tool to support person-centred communication in memory clinics by Tanja J de Rijke, Kyra KM Kaijser, Dianne Vasseur, Hilal Tasköprü, Lotte Huisman, Aniek M van Gils, Vera Otten, Carolien Smits, Cynthia S Hofman, Minke Kooistra, Ellen MA Smets, Thomas Engelsma and Leonie NC Visser in DIGITAL HEALTH

sj-pdf-3-dhj-10.1177_20552076251412631 - Supplemental material for Design and development of ‘Helder in Gesprek’: A tool to support person-centred communication in memory clinics Supplemental material, sj-pdf-3-dhj-10.1177_20552076251412631 for Design and development of ‘Helder in Gesprek’: A tool to support person-centred communication in memory clinics by Tanja J de Rijke, Kyra KM Kaijser, Dianne Vasseur, Hilal Tasköprü, Lotte Huisman, Aniek M van Gils, Vera Otten, Carolien Smits, Cynthia S Hofman, Minke Kooistra, Ellen MA Smets, Thomas Engelsma and Leonie NC Visser in DIGITAL HEALTH

## List of abbreviations

SCD: Subjective Cognitive Decline
MCI: Mild Cognitive Impairment
UX: User eXperience
Lo-fi: low-fidelity
TAM: Technology Acceptance Model
NPS: Net Promoter Score
FCCHL: Functional Communicative and Critical Health Literacy scale
DHLI: Digital Health Literacy Instrument
PrEmo: Product Emotion Measurement Instrument
