# Supplementary material for: Design and development of ‘Helder in Gesprek’: A tool to support person-centred communication in memory clinics
Source: Digit Health. 2026 Jan 20;12:20552076251412631. doi: 10.1177/20552076251412631 (PMC12820018; doi:10.1177/20552076251412631)
Supplement: sj-docx-1-dhj-10.1177_20552076251412631 - Supplemental material for Design and development of ‘Helder in Gesprek’: A tool to support person-centred communication in memory clinics [file sj-docx-1-dhj-10.1177_20552076251412631.docx]

Supplementary materials for manuscript: ‘Design and development of ‘Helder in Gesprek’: a tool to support person-centred communication in memory clinics’

## Supplement 1: Positionality statement and reflexive practice

Positionality statement:
As a Dutch female PhD researcher trained in health and social sciences, I (TR) approach this project from a transformative research paradigm with a focus on human-centred participatory design and acknowledge the importance of the lived experience of people with cognitive complaints. I will reflect throughout the research process after every co-research or co-design session and halfway throughout the User eXperience (UX) and usability tests individually and within the facilitating team to ensure that the process and development of the tool is done in an ethical way and accurately contains the needs and preferences of those it is meant to serve. To the best of my possibilities, I will acknowledge and address pre-existing power dynamics of the entity of academic research as much as possible within this project, for instance, by engaging in recruitment in community settings, showing up as a ‘human being’ myself and explaining my personal motivation, offering multiple ways to participate (e.g. by offering multiple materials that are not too aesthetically appealing so people might be hesitant to work with it), allotting time for informal chatting, and rapport building, and addressing creative and intellectual self-trust via creating success experiences via exercises where there are no right or wrong answers.

Reflexive practice:

In this project, we continuously reflected on our practice throughout the process. During the co-research, co-design, and usability and UX sessions, one facilitator took notes and watched for any signs of distress, both verbal and non-verbal, among the people involved. This helped us stay alert to how participants are feeling, ensuring their well-being and maintaining a respectful, supportive environment. After each session, the facilitators engaged in a quick debrief including a critical reflection on the process. When a participant became distressed, we temporarily diverted the attention from the group away from that person, offered a break and discussed the situation with the person in distress. When relevant and desired, we discussed the issue that caused distress with the entire group. Ethical challenges encountered during the study comprised epistemic justice (how do we weigh and value different types of knowledges?), concrete versus providing space (how to make the sessions as concrete as possible for people with cognitive complaints, whilst providing enough space for bottom-up/unexpected input along the way), role of a researcher (how personal do you get with co-researchers and co-designers? What is your role and responsibility as a researcher?), power differences (How to deal with power differences between group members within a co-design group?), identity disclosure versus privacy (what needs to be shared in a positionality and reflexivity statement versus how much do you want to share about yourself online), inclusivity (who was able to join the study and who was excluded), relationship building (how to ‘exit’ a co-design process and its relationships in a good way), and time (how to navigate taking time for the process and having a PhD deadline). We had regular meetings with a sounding board, comprising professionals with expertise in dementia, medical psychology, medical communication, human-centred design, health innovation & implementation, and had two critical friends specialized in participatory and inclusive research. These meetings provided us with a safe space to openly discuss what approach seems to work or not,  and any biases, ethical challenges, or limitations we might have. This ongoing process of reflection was fundamental to understanding our own roles and how we could adapt in real-time, ultimately improving the co-design process and making it more inclusive and responsive.

Throughout the entire study, the Double Diamond model was used as a framework. We experienced this framework as helpful to explain the structure of the current study to the co-researchers and co-designers, especially to those with cognitive complaints. We also consciously celebrated the end of each phase with co-researchers or co-designers to signpost an achievement and transition to the next phase. Due to the neurodegenerative nature of the disease, the limited resources available, and the need for diversity, we could not work with the same people in the same role throughout the entire project resulting in people switching roles after each phase (e.g. moving from a co-research or co-design role towards a participant role during user testing; see supplement 17). This way, people could still be involved in the project, yet in a different way. At the beginning of each phase, we asked people what location and time they preferred, to ensure that the environment as was comfortable as possible. In phase I, sessions took 60 minutes (as recommended by the coordinator from the community centre who knew the co-researchers personally) and in phase II sessions took 90 minutes. We extended the timing in phase II, because we learned from phase I that 60 min is very short to get in-depth group discussions and insights, while also ensuring enough time for rapport building within the team and offering sufficient breaks. Sessions contained a warm-up/opening part, two exercises, and a closing. We selected exercises that matched the overarching goal of the project, such as gain insight from all stakeholders, which is similar to standard design practices.

Following this, we carefully considered how to adapt our methods to effectively gather this information in a comfortable and fun way, especially given the cognitive challenges of some group members (which may also fluctuate per day or even during a session). This approach allowed us to remain goal-oriented while tailoring our activities to ensure meaningful and stress-free participation from people with cognitive impairments, for instance, in the form of creative exercises or via visual materials.

Visual materials and methods worked well when collaborating with people with cognitive complaints. Not only to visually explain the question, but also for people to explain themselves and answer in nuanced ways. For instance, we got more nuanced answers during UX testing when using the product emotion measurement (PrEmo) than when asking an open question. Another example is that drawing a question sometimes also helped to understand the question more clearly and steer conversations (consciously and unconsciously) back to the topic of discussion. We offered multiple materials and forms for visual participation and to address any expectations that someone’s contribution must be artistic or visually appealing. In our co-design group, we did so by doing small ‘warm-up’ exercises focussing on relationships and ‘making something’ together by adding limitations so that the end-product is not likely to end up beautiful and therewith the unwanted pressure to create something beautiful is minimized (e.g. time limitations so that there is no time to think about what you are creating, finishing the creation of your neighbour, or adding physical limitations such as close your eyes).

Another element that we addressed and took into account when selecting exercises was self-trust. We tried to increase self-trust as much as possible by creating a respectful environment, offering explicit recognition and acknowledgement, and introducing ‘successful’ experiences from the beginning (e.g. by having people pick a card with a drawing that best resembles the experience of having cognitive complaints to them). We also framed the project as a ‘let’s figure it out together’ mentality, that offered space for exploration and decreased people’s worries about their cognitive ability.

**Supplement 2: Visual flow overview of people involved in phase I, phase II, and phase III**


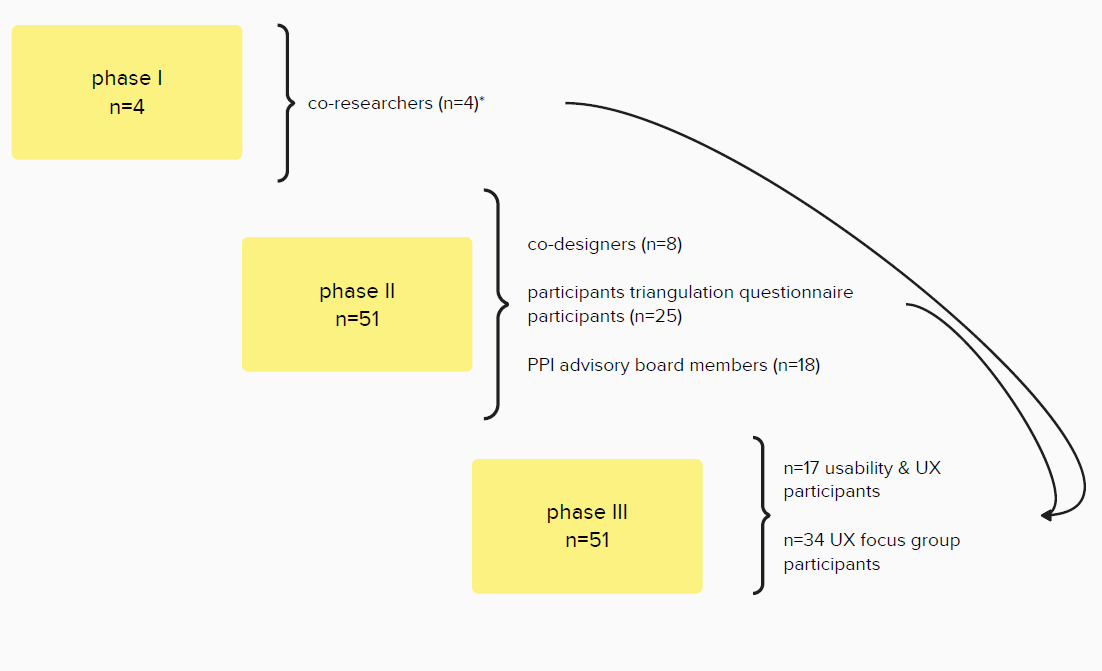


**Supplementary figure 2. Visual flow overview of people involved throughout the Helder in Gesprek study.**
*Notes. *One co-researcher dropped out half-way during personal circumstances.*

## Supplement 3: COnsolidated criteria for REporting Qualitative research (COREQ) guidelines

***insert separate PDF***

## Supplement 4: Information on phase I: problem statement

Overview of sessions

Phase I started with a non-binding recruitment meeting to get to know each other, including personal reasons for doing the research, and to explain the project. During the meeting, people conducted a small photo-elicitation assignment that matched their interpretation of ‘cognitive complaints’. Herewith, potential co-researchers got a feeling of what the project entailed, got used to sharing their opinion and ideas with the group, and induced a feeling of self-efficacy regarding being a co-researcher. After this introduction meeting, people could decide if they wanted to be a co-researcher or not. All interested people (n=4; see Figure 1) wanted to continue and become a co-researcher.


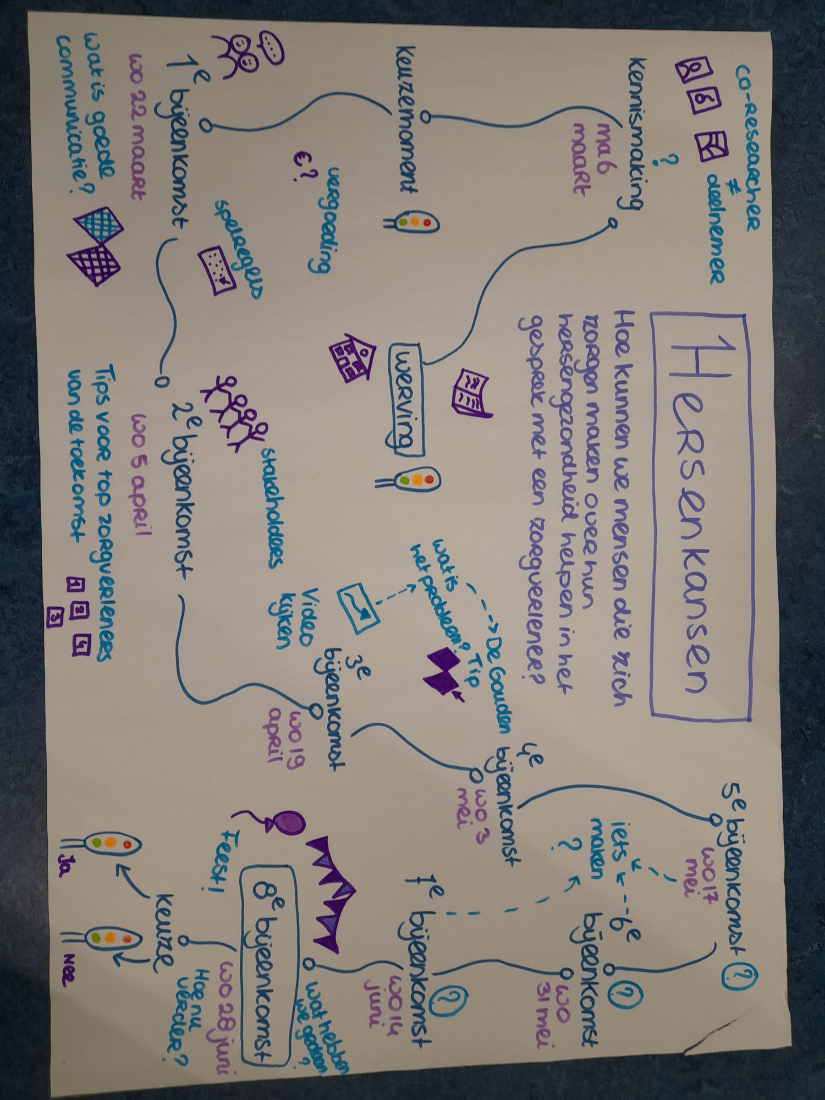
The co-research trajectory was not predetermined, leaving ample room for iterative insights during the co-research trajectory. To provide the team (facilitators n=3; co-researchers n=4) with some structure throughout the project, we created a roadmap (see supplementary Figure 1), which we gradually filled in.

**Supplementary figure 1 - Roadmap of phase I. Roadmap contains the title of the group, the research question, dates and number of the session (e.g. 2^nd^ session Wednesday 5 April) and brief descriptions of the topic of the sessions (see supplementary Table 1).**

The underlying structure of this roadmap was based on the first diamond of the Double Diamond model^1^, containing the ‘discover’ phase (i.e. understanding all relevant problems, causes, and contexts) and the ‘define’ phase (i.e. combining what one has discovered so far into one problem definition). Thematic content of the other sessions can be found below in supplementary Table 1.

**Supplementary Table 1. Thematic content of the co-research sessions in phase I.**

| **Sessions** | **Overarching theme of the session** |
| --- | --- |
| Session 1 | What is good communication? |
| Session 2 | Tips & tops regarding communication for clinicians of the future |
| Session 3 | Perspectives on different communication styles and strategies |
| Session 4 | The golden tip: what is the problem that needs solving from the perspective of co-researchers |
| Session 5 | Diving deeper into the context of the golden tip: What is the problem behind the key problem? Who is involved? |
| Session 6 | 5Ws: When do we need to solve this? What helps? What doesn’t help? What do clinicians need to know? With what do they need to know? |
| Session 7 | Characteristics of a potential solution |
| Session 8 | Reflection, celebrating together, writing a newsletter item, and how we will move on from here |

Sessions were facilitated and prepared by three facilitators (DV, HT, TR) leaving ample room for changes/flexibility during the session when needed. The sessions lasted on average 60 min and took place in a separate room of a specific community centre, which was the preferred location by the participants. Every meeting started off with informal chatting, followed by signposting based on the roadmap (e.g. what is the project about, what are we doing, why are we doing what we are doing, where are we going, what will we be the topic of today etc.) with ample room for questions and any practicalities. Sessions usually comprised one extensive exercise or two smaller exercises, in which decisions regarding the project were made collaboratively (i.e. on what problem exactly needs to be solved). Breaks were included, when needed. The last five to ten minutes always comprised a recap of the session, handing out reimbursements, any practicalities, room for questions, and handing out written summaries of the session for people to read and share with loved ones.

Example exercise

An example exercise is ‘The Golden Tip’ from session 4. We provided duo’s of participants with a flip over which contained all main problems with person-clinician communication in the memory clinic as inventoried in a previous session. We asked the duo’s to map the problems on the flip over in one of their pre-indicated sections: not important, a bit important, and very important. Every section should at least contain one problem, to avoid all problems ending up in the section ‘very important’ part. Hereafter, we asked participants to fold the flip over, such that only the section ‘very important’ could be seen. Next, we asked participants to rank the items qualified as ‘very important’ from 1 (most important to solve) to 3 (least important to solve), resulting in a top 3 per duo. Results were discussed with the group to reach consensus regarding one problem that most urgently needs to be solved , i.e., the golden tip.

Facilitators did not participate in these exercises or group discussions, but made field notes and provided assistance when needed.

## Supplement 5: Information on phase II: co-design sessions

Overview of sessions

Similar to phase I, the co-design trajectory in phase II was not predetermined to leave room for emerging insights during the trajectory, while providing the team (facilitators n=3; co-designers n=8) some structure by using a roadmap. The underlying structure of this roadmap was mainly based on the second diamond of the Double Diamond model^1^, containing the ‘develop’ phase (i.e. brainstorm, experiment and test multiple solutions) and the ‘deliver’ phase (i.e. focus onto the best solution and hone it). Since phase II involved new participants, we with some exercises touching upon the ‘define’ phase of the first double diamond to validate our findings from phase I (i.e. combining what one has discovered so far into one problem definition). From session 4 onwards, a UX designer from design agency Kaliber provided advice on the content of the sessions. Thematic content of the other sessions can be found below in Supplementary Table 2.

**Supplementary Table 2. Thematic content of the co-research sessions in phase II.**

| **Sessions** | **Overarching theme of the session** |
| --- | --- |
| Session 1 | Getting to know each other + what does communication in the memory clinic look like now, in a dream scenario in the future, and how do we get there? |
| Session 2 | What would you want your memory clinic clinician to know about you + characteristics of a solution |
| Session 3 | What are potential solutions, what are good elements of potential solutions, and how can we combine them into a new solution? |
| Session 4 | Experience journey mapping |
| Session 5 | Preferences for the medium of the solution, information needs and actual information provision throughout the patient journey, and dream outcomes of the solution |
| Session 6 | Big update, evaluation, and how will we move on from here |


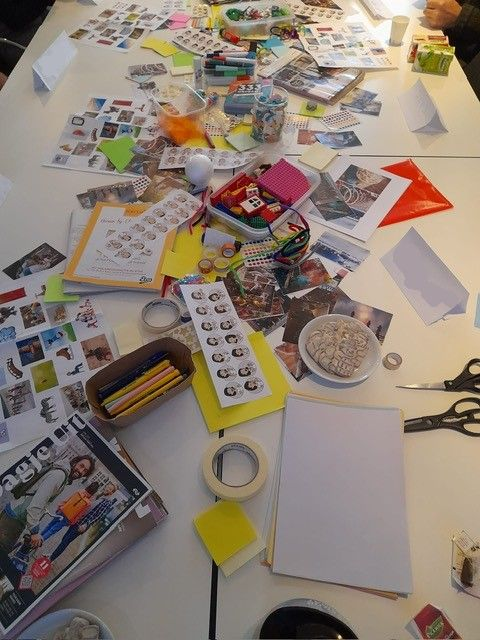
Sessions were facilitated and prepared by three facilitators (KK, HT, TR) leaving ample room for changes/flexibility during the session. The sessions lasted on average 90 min. For the first meeting, we spent a lot of time on introductions, including everyone’s motivation to be involved in the research. Every meeting started with informal chatting, followed by signposting based on the roadmap (e.g. what is the project about, what are we doing, why are we doing what we are doing, where are we going, what will we be the topic of today ) with room for questions, and any practicalities. Hereafter, we conducted a ‘warming up’ exercise to facilitate group building, creative and associative thinking, and get into a ‘making/doing’ mode. We consciously did this every session to lower the barrier for making/creating things as much as possible and to remind participants that the session was not about being artistically gifted, but rather about the process to create a solution and thoughts behind the making process. Sessions usually comprised two smaller exercises, either individually or in small groups. When needed, memory clinic clinicians received a separate exercise related to their professional role in the memory clinic. After each exercise, results were shared with the group and consensus was sought, if needed. The last five to ten minutes always comprised a recap of the session, handing out reimbursements, any practicalities, and room for questions. A summary containing the content and results of each session was e-mailed after every session.

**Supplementary Figure 2 - Materials for session 3, including the 'Pass me on' exercise.**

Example warming-up exercise

An exemplary warming-up exercise is the ‘Pass me on’ exercise from session 3. During the ‘Pass me on’ exercise participants have to make something with the various materials provided on the table (see supplementary Figure 2), such as pictures, magazines, 3D shapes, toy cars, stickers, feathers, clay, tape, LEGO and Playmobil, to stimulate creativity. Participants had only 30 seconds to create something, to minimize doubts about what to create and the pressure to make something beautiful. The participants had to do all this in silence. After 30 seconds, they were asked to pass their creation on to their neighbour who had to further the creation. We did so until the creation was back at its initial creator. The underlying goal behind this exercise was to stimulate creation, creative thinking, and building upon each other’s ideas.

Example exercise


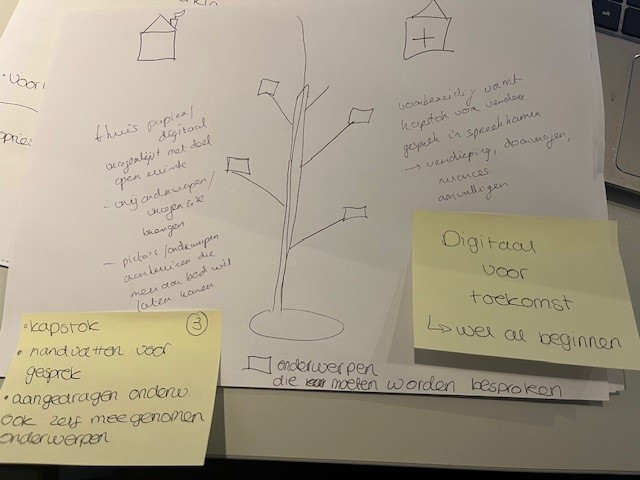
Another exemplary exercise from session 3 was the ‘CRAZY 4’s and prototype remix’ exercise. The exercise started off individually. Participants were given 15 minutes to fold an empty A4 paper in four quadrants and to sketch four completely different solutions for the problem: *‘What does a tool look like that helps the memory clinic clinician to get to know the patient better?’* Memory clinic clinicians were asked to perform the exercise from a memory clinic clinician perspective. Next, the group was divided in three groups of one person with cognitive complaints, one care partner, and one clinician. We asked the groups to explain their CRAZY 4’s to each other and comment on what they liked and disliked about the designs. Hereafter, each trio had to come up with a final solution, containing elements from their individual CRAZY 4’s (=prototype remix; see supplementary Figure 3). These three final solutions were then shared and discussed among the entire group.

**Supplementary Figure 3 - Example of a combined prototype remix result of a trio.**

**Supplement 6: Phase II: Triangulation questionnaire people with cognitive complaints**

1. **I am a:**
   1. Man
   2. Woman
   3. Other, namely: …
   4. I prefer not to tell
2. **What is your age in years?**
3. **What level of education did you finish last?**
   1. Practical education
   2. Preparatory vocational education
   3. Senior general secondary education
   4. Pre-university secondary education
   5. Higher professional education
   6. University
   7. Other, namely: …
4.
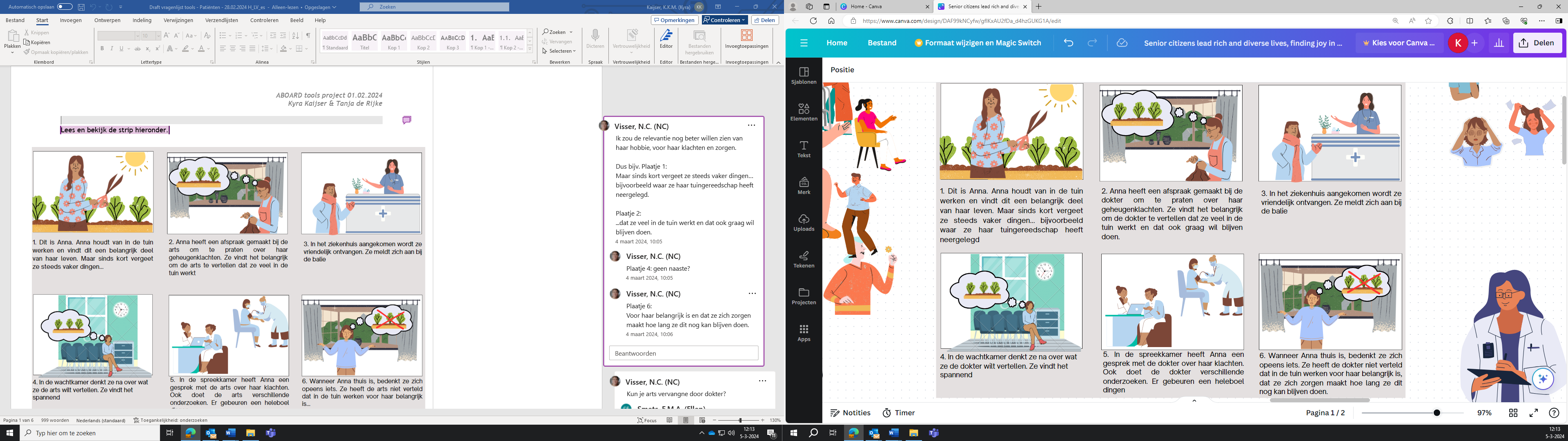


**The cartoon above shows Anna's story. Anna went to the memory clinic in the hospital. She wanted to tell the doctor something about her life, so that the doctor knows what is important to her and can take this into account. Telling the doctor in hospital about your own life and personal situation is important, but can sometimes be difficult. For example, because you don't remember exactly what you wanted to ask or tell and it can be difficult to find the right moment. Anna wants to tell during her appointment with the doctor what is important to her - gardening. Do you think it is important for the doctor to know something about your personal life?**

1. Very important
2. Important
3. Not important and important
4. Not important
5. Definitely not important
6. **Could you please explain your answer [to question 4]?**
7. **Yet Anna was unable to tell the doctor that gardening was important to her. Do you recognise this, that you would have liked to tell something but were unable to?**
   1. Definitely yes
   2. Yes
   3. A bit yes and a bit not
   4. No
   5. Definitely not
8. **Could you please explain your answer [to question 6]?**
9. **In the comic, you saw the story of Anna. Anna finds it important to tell the doctor that she likes to work in the garden. What are topics that are important for you to tell a doctor at the memory clinic?** [multiple answers possible]
   1. Which people are important to me
   2. That I live alone or with others
   3. That I take care of others
   4. That I would like to live at home for as long as possible
   5. That I am retired or just that I am still working
   6. That I would like to prepare for the future
   7. That I talk about my worries easily or with difficulty
   8. That I am afraid of the future
   9. That I find it difficult to cope with my memory problems
   10. That I have a busy social life
   11. That I cope well or not well with stimuli
   12. What my hobbies are and other activities that make me feel good
   13. That I have a bad feeling because some things no longer work out
   14. That I am afraid that I won't be able to do things on my own in the future.
   15. Nothing from this list.
10. **Are there any other personal things a doctor should know about (what is important to) you?** [open question]
11. **The GP or another doctor refers you to the memory clinic. You make an appointment and prepare at home. At the memory clinic, you will be welcomed in the waiting room. Then you will have an interview with a doctor. These steps are also called the patient journey (see Figure 1). During this patient journey, there are several moments when you can be occupied with what you want to tell the doctor or other healthcare providers. For example, when you are thinking at home about the questions you have for the doctor, or during the conversation with the doctor in which you tell your story. The following questions are about the patient journey. If you are thinking about preparing for the visit to the memory clinic at home, and you are thinking about the topics you would like to tell the doctor: What helps you think about what you would like to tell the doctor?** [open question]
12. **What makes it difficult to think of what you want to tell the doctor?** [open question]
13. **When you think about the waiting room at the memory clinic, and you think about the topics you would like to tell the doctor: What helps you think of what you would like to tell the doctor?** [open question]
14. **What makes it difficult to think of what you would like to tell the doctor?** [open question]
15. **When you think about talking to the doctor at the memory clinic, and you think about the topics you would like to tell the doctor: What helps you tell it?** [open question]
16. **What makes it difficult to tell?** [open question]
17. **We understand that telling a doctor about topics that are important to you or your loved one can be difficult. Yet it is important for you, your loved one and the doctor to do. That is why we are creating a tool to help with this. The tool is currently still under development. Therefore, we are now asking you some questions about the desired shape and features of a tool. What kind of tool could this be?** [open question]
18. **What does the tool to be designed look like?** [multiple answers possible]
    1. Something digital (like an app or website)
    2. Something on paper
    3. Something 3D
    4. Other, namely: ….
19. **What does the tool to be designed contain?** [multiple answers possible]
    1. Something text-based
    2. Something with images/pictures
    3. Something with audio
    4. Other, namely: …
20. **When does the tool to be designed get used?** [multiple answers possible]
    1. Something that you can use at home
    2. Something that you can use in the memory clinic
    3. Other, namely: ….
21. **Who uses the tool to be designed?** [multiple answers possible]
    1. To be used by yourself
    2. To be used together with someone else (such as your care partner)
    3. To be used together with the clinician
    4. Other, namely: …
22. **How often should you be able to use the tool?** [multiple options possible]
    1. Something that you can use once
    2. Something that you can use multiple times
    3. Other, namely: …
23. **Do you have any additional ideas on what a tool should look like or how you would like to use it? [**open question]

**Supplement 7: Phase II: Triangulation questionnaire care partners**

1. **I am a:**
   1. Man
   2. Woman
   3. Other, namely: …
   4. I prefer not to tell
2. **What is your age in years?**
3. **What level of education did you finish last?**
   1. Practical education
   2. Preparatory vocational education
   3. Senior general secondary education
   4. Pre-university secondary education
   5. Higher professional education
   6. University
   7. Other, namely: …
4. **You are receiving this questionnaire because you are a care partner of someone who has been to the memory clinic. Did you attend the appointment at the memory clinic?**
   1. Yes
   2. No
   3. Sometimes
   4. I do not know
5.
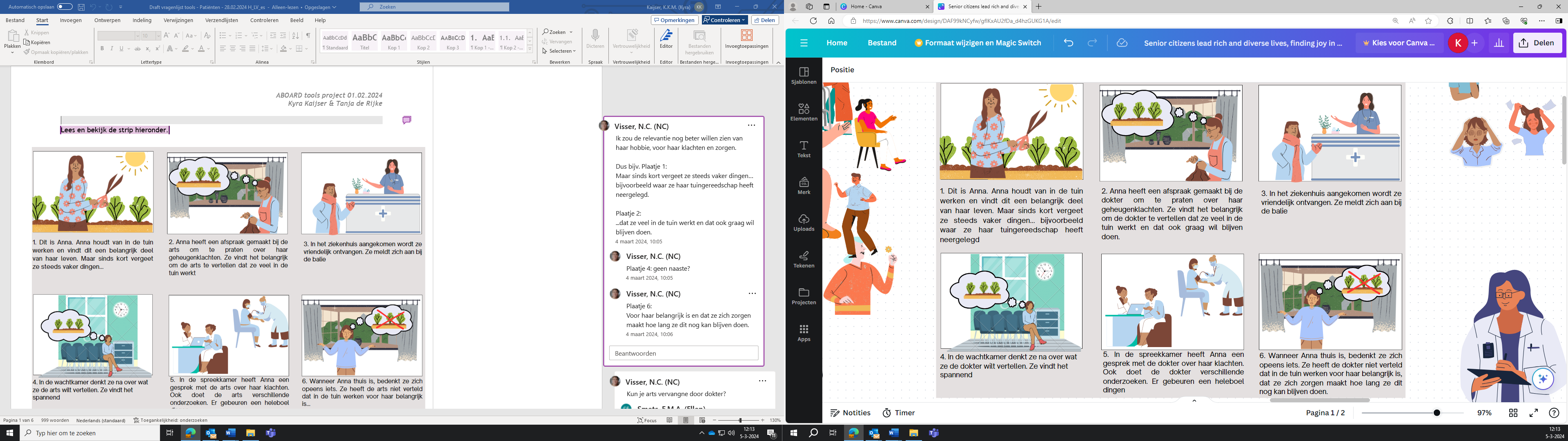


**The cartoon above shows Anna's story. Anna went to the memory clinic in the hospital. She wanted to tell the doctor something about her life, so that the doctor knows what is important to her and can take this into account. Telling the doctor in hospital about your own life and personal situation is important, but can sometimes be difficult. For example, because you don't remember exactly what you wanted to ask or tell and it can be difficult to find the right moment. Anna wants to tell during her appointment with the doctor what is important to her - gardening. Do you think it is important for the doctor to know something about your (or that of your loved one) personal life?**

1. Very important
2. Important
3. Not important and important
4. Not important
5. Definitely not important
6. **Could you please explain your answer [to question 4]?**
7. **Yet Anna was unable to tell the doctor that gardening was important to her. Do you recognise this, that you would have liked to tell something (or about your loved one) but were unable to?**
   1. Definitely yes
   2. Yes
   3. A bit yes and a bit not
   4. No
   5. Definitely not
8. **Could you please explain your answer [to question 6]?**
9. **In the comic, you saw the story of Anna. Anna finds it important to tell the doctor that she likes to work in the garden. What are topics that are important for you to tell a doctor at the memory clinic?** [multiple answers possible]
   1. Which people are important to my loved one
   2. That my loved one lives alone or with others
   3. That my loved one takes care of others
   4. That my loved one would like to live at home for as long as possible
   5. That my loved one is retired or just that I am still working
   6. That my loved one would like to prepare for the future
   7. That my loved one talks about my worries easily or with difficulty
   8. That my loved one is afraid of the future
   9. That my loved one finds it difficult to cope with my memory problems
   10. That my loved one has a busy social life
   11. That my loved one copes well or not well with stimuli
   12. What my loved one’s hobbies are and other activities that make him/her feel good
   13. That my loved one has a bad feeling because some things no longer work out
   14. That my loved one is afraid that he/she won't be able to do things on his/her own in the future.
   15. Nothing from this list.
10. **Are there any other personal things a doctor should know about (what is important to) you? [**open question]
11. **The GP or another doctor refers you to the memory clinic. You make an appointment and prepare at home. At the memory clinic, you will be welcomed in the waiting room. Then you will have an interview with a doctor. These steps are also called the patient journey (see Figure 1). During this patient journey, there are several moments when you can be occupied with what you want to tell the doctor or other healthcare providers. For example, when you are thinking at home about the questions you have for the doctor, or during the conversation with the doctor in which you tell your story. The following questions are about the patient journey. If you are thinking about preparing for the visit to the memory clinic at home, and you are thinking about the topics you would like to tell the doctor: What helps you think about what you would like to tell the doctor?** [open question]
12. **What makes it difficult to think of what you want to tell the doctor?** [open question]
13. **When you think about the waiting room at the memory clinic, and you think about the topics you would like to tell the doctor: What helps you think of what you would like to tell the doctor?** [open question]
14. **What makes it difficult to think of what you would like to tell the doctor?** [open question]
15. **When you think about talking to the doctor at the memory clinic, and you think about the topics you would like to tell the doctor: What helps you tell it?** [open question]
16. **What makes it difficult to tell?** [open question]
17. **We understand that telling a doctor about topics that are important to you or your loved one can be difficult. Yet it is important for you, your loved one and the doctor to do. That is why we are creating a tool to help with this. The tool is currently still under development. Therefore, we are now asking you some questions about the desired shape and features of a tool. What kind of tool could this be?** [open question]
18. **What does the tool to be designed look like?** [multiple answers possible]
    1. Something digital (like an app or website)
    2. Something on paper
    3. Something 3D
    4. Other, namely: ….
19. **What does the tool to be designed contain?** [multiple answers possible]
    1. Something text-based
    2. Something with images/pictures
    3. Something with audio
    4. Other, namely: …
20. **When does the tool to be designed get used?** [multiple answers possible]
    1. Something that you can use at home
    2. Something that you can use in the memory clinic
    3. Other, namely: ….
21. **Who uses the tool to be designed?** [multiple answers possible]
    1. To be used by yourself
    2. To be used together with someone else (such as your care partner)
    3. To be used together with the clinician
    4. Other, namely: …
22. **How often should you be able to use the tool?** [multiple options possible]
    1. Something that you can use once
    2. Something that you can use multiple times
    3. Other, namely: …
23. **Do you have any additional ideas on what a tool should look like or how you would like to use it?** [open question]

**Supplement 8: Phase II: Triangulation questionnaire clinicians**

1. **I am a:**
   1. Man
   2. Woman
   3. Other, namely: …
   4. I prefer not to tell
2. **What is your age in years?**
3. **What is your profession?**
   1. Neurologist
   2. AIOS neurology
   3. ANIOS neurology
   4. Geriatrician
   5. AIOS geriatrics
   6. ANIOS geriatrics
   7. Psychiatrist
   8. AIOS psychiatry
   9. ANIOS psychiatry
   10. Neuropsychology
   11. Specialized nurse
   12. Nurse
   13. Other, namely: …
4. **How important do you think it is for the quality of care you provide at the memory clinic to know about a patient's personal situation and social environment and things that are important to them, such as their social environment, daily activities or hobbies, in addition to (bio)medical background and current symptoms?**
   1. Not at all important
   2. Not important
   3. A bit not important
   4. A bit important and not important
   5. A bit important
   6. Important
   7. Very important
5. **Could you please elaborate [on your answer at question 4]?**
6. **Patients and loved ones indicate that they find it important to give the caregiver(s) at the memory clinic a complete picture of their personal situation and things that are important to them, such as their needs, social environment, and activities or hobbies. By sharing this information, patients feel seen and heard as human beings. In your daily practice, to what extent do you, as a clinician, experience the opportunity to get such a complete picture of a patient?**
   1. I see no opportunity at all
   2. I see no opportunity
   3. I see not really an opportunity
   4. I see not really an opportunity and a bit an opportunity
   5. I see a bit an opportunity
   6. I see an opportunity
   7. I definitely see an opportunity
7. **Could you please elaborate [on your answer at question 6]?**
8. **It can be difficult for patients and their loved ones to raise their personal situation and what is important to them when talking to a healthcare provider. We are currently working with patients, loved ones and healthcare providers to create a tool to support patients and their loved ones to do this appropriately and efficiently. What are your views on the development of such a tool?**
   1. Definitely not useful
   2. Not useful
   3. A bit not useful
   4. A bit not useful and a bit useful
   5. A bit useful
   6. Useful
   7. Definitely useful
9. **Could you please elaborate [on your answer at question 8]?**
10. **What do you think the use of such a tool could benefit the patient?** [open question}
11. **What do you think the use of such a tool could benefit you as a caregiver at the memory clinic?** [open question]
12. **When developing the tool, it is important to think about future implementation in good time. The tool is currently still under development. Therefore, we are now asking you some questions about the desired form and features of such a tool. In other words, we are talking about a tool that will better enable patients and loved ones to give caregiver(s) at the memory clinic a complete picture of their personal situation and things that are important to them, such as their needs, social environment, and daily activities or hobbies. What features do you think this tool should have?** [open question]
13. **What does the tool to be designed look like?** [multiple answers possible]
    1. Something digital
    2. Something on paper
    3. Something 3D
    4. Other, namely: ….
14. **What does the tool to be designed contain?** [multiple answers possible]
    1. Something text-based
    2. Something with images/pictures
    3. Something with audio
    4. Other, namely: …
15. **When does the tool to be designed get used?** [multiple answers possible]
    1. Something that the patient (and care partner) have prepared at home
    2. Something that you, as clinician, can fill in together with the patient (and care partner) during the consultation
    3. Something that you, as clinician, can use together with the patient (and care partner) during the consultation
    4. Other, namely: ….
16. **The tool to be designed is based on the input of:**  [multiple answers possible]
    1. Only based on input by the patient
    2. Input by the patient and his/her care partner
    3. Other, namely: ….
17. **How does the tool to be designed get used?** [multiple answers possible]
    1. Something that you, as clinician, can use as a preparation for the consultation
    2. Something that you, as clinician, can use as reference work
    3. Something that the patient can use as preparation for the consultation
    4. Something that the patient can use as reference work
    5. Other, namely: …
18. **Suppose a tool becomes available to get a more complete picture of patient visiting the memory clinic: as a clinician, what would drive/help/facilitate you to use this tool?** [open question]
19. **Suppose a tool becomes available to get a more complete picture of patient visiting the memory clinic: as a clinician, what would limit/hinder you to use this tool?** [open question]
20. **What type of clinician should use such a tool in the memory clinic?** [multiple answers possible]
    1. Neurologist
    2. AIOS neurology
    3. ANIOS neurology
    4. Geriatrician
    5. AIOS geriatrics
    6. ANIOS geriatrics
    7. Psychiatrist
    8. AIOS psychiatry
    9. ANIOS psychiatry
    10. Neuropsychology
    11. Specialized nurse
    12. Nurse
    13. Other, namely: …
21. **How much time do you have in clinical practice to offer personalised attention to a patient other than medical aspects?**
    1. 0-5 minutes
    2. 6-10 minutes
    3. 11-15 minutes
    4. 16-20 minutes
    5. 21-25 minutes
    6. 26-30 minutes
    7. Other, namely: …
22. **What should be the maximum amount of time that the use of the tool should take you as a healthcare provider in talking to a patient and loved one?**
    1. 0-5 minutes
    2. 6-10 minutes
    3. 11-15 minutes
    4. 16-20 minutes
    5. 21-25 minutes
    6. 26-30 minutes
    7. Other, namely:
23. **Who do you think should be responsible for introducing the tool to people visiting the memory clinic?**
    1. General practitioner
    2. Clinicians at the memory clinic
    3. Front desk assistants at the memory clinic
    4. Societal organisations
    5. Patient organisations
    6. Healthcare insurers
    7. Other, namely: …
24. **Who do you think should be responsible for the maintenance of the tool?**
    1. General practitioner
    2. Clinicians at the memory clinic
    3. Front desk assistants at the memory clinic
    4. Societal organisations
    5. Patient organisations
    6. Healthcare insurers
    7. Other, namely: …
25. **Who do you think should be responsible for the costs of the tool?**
    1. General practitioner
    2. Clinicians at the memory clinic
    3. Front desk assistants at the memory clinic
    4. Societal organisations
    5. Patient organisations
    6. Healthcare insurers
    7. Other, namely: …
26. **If you have to make an estimate, what should be the maximum cost of this device per patient?**
    1. 1-5 euro
    2. 6-10 euro
    3. 11-15 euro
    4. 16-20 euro
    5. 21-25 euro
    6. 26-30 euro
    7. Other, namely: ….

**Supplement 9: Phase II: results triangulation questionnaire**

**Supplementary Table 3. Study sample triangulation questionnaire**

| **Person with cognitive complaints (n=7)** |  |
| --- | --- |
| Age, mean | 66.4 (SD= 8.6) years |
| Gender | 57% female |
| Educational attainment* | Low: 11.1%  Medium: 22.2%  High: 66.7% |
| Do you think it is important that a clinician knows something about your personal life? | 7.71±2.81 (range: 2-10; min=0 not important at all; max=10 very important) |
| Do you ever feel like you wanted to tell the doctor something, but did not? | 4.57±3.16 (range: 0-8; min=0 definitely not; max=10 definitely yes) |
| Themes that I would like to discuss** | Who is important to me: 57.1%  That I live by myself or with others: 57.1%  That I have care responsibilities of others: 14.3%  That I would like to live at home for as long as possible: 85.7%  That I am working or retired: 42.9%  That I would like to prepare myself for the future: 28.6%  That I easily talk about my concerns: 42.9%  That I am afraid of the future: 28.6%  That I have difficulty coping with my cognitive complaints: 57.1%  That I have a busy social life: 14.3%  That I cannot handle external stimuli well: 14.3%  What my hobbies/other activities are that make me feel good: 42.9%  That I do not feel great emotionally, because certain things are not possible anymore: 42.9%  That I am afraid to become dependent in the future: 85.7% |
| Preferred characteristics of the tool** | Digital: 85.7%  Paper-based: 28.6%  Text-based: 85.7%  Image/picture-based: 28.6%  Speech-to-text: 42.9%  To be used at home: 85.7%  To be used in the memory clinic: 57.1%  To be used by me only: 42.9%  To be used by my care partner: 42.9%  To be used by me and my care partner: 42.9%  To be used by me, my care partners, and my clinician: 28.6%  To be used multiple times: 85.7% |
| **Care partner (n=6)** |  |
| Age, mean | 60.5 (SD=10.4) years |
| Gender | 100% female |
| Educational attainment* | Low: 16.7%  Medium: 50%  High: 33.3% |
| You are receiving this questionnaire because you are a care partner of someone who has been to the memory clinic. Did you attend the appointment at the memory clinic? | Yes: 100% |
| Do you think it is important that a clinician knows something about your loved one’s personal life? | 8.17±1.33 (range: 6-10; min=0 not important at all; max=10 very important) |
| Do you ever feel like you or your loved one wanted to tell the doctor something, but did not? | 3.33±3.78 (range: 0-8; min=0 definitely not; max=10 definitely yes) |
| Themes that my loved one wants to discuss** | Who is important to my loved one: 83.3%  That my loved one lives by him/herself or with others: 50%  That my loved one would like to live at home for as long as possible: 66.7%  That my loved one is working or retired: 50%  That my loved one would like to prepare him/herself for the future: 33.3%  That my loved one easily talks about his/her concerns: 66.7%  That my loved one is afraid of the future: 50%  That my loved one has difficulty coping with his/her cognitive complaints: 33.3%  That my loved one cannot handle external stimuli well: 50%  What the hobbies/other activities are that make my loved one feel good: 33.3%  That my loved one does not feel great emotionally, because certain things are not possible anymore: 83.3%  That my loved one is afraid to become dependent in the future: 66.7% |
| Preferred characteristics of the tool** | Digital: 66.7%  Paper-based: 83.3%%  3D: 16.6%  Text-based: 50%  Image/picture-based: 50%%  Speech-to-text: 50%  To be used in the memory clinic: 33.3%  To be used by me, my care partners, and my clinician: 100%  To be used once: 83.3%  To be used multiple times: 100% |
| **Clinician (n=12)** |  |
| Age, mean | 35.6 (SD=10.7) years |
| Gender | 83.3% female |
| Profession | Neurologist: 8.3%  ANIOS Neurology: 25%  Geriatrician: 16.7%  Neuropsychologist: 25%  Specialized nurse: 8.3%  Nurse: 8.3%  Other***: 16.7% |
| How important is it for the quality of care to have a comprehensive understanding of someone’s situation? | 8.5±0.8 (range: 7-10; min=0 not at all important; max=10 very important) |
| As a clinician, to what extent to you experience in your daily practice the opportunity to get such as comprehensive understanding of someone’s situation? | 7.08±1.17 (range: 5-9; min=0 no opportunity at all; max=10 definitely opportunity) |
| How do you feel about the development of a tool that supports people to share about their personal situation and bring up topics that they find important to discuss with you? | 8.33±0.99 (range: 7-10; min=0 definitely not useful; max=10 definitely useful) |
| Preferred characteristics of the tool** | Digital: 83.3%  Paper-based: 91.7%  3D: 25%  Text-based: 83.3%  Image/picture-based: 83.3%  Speech-to-text: 25%  People visiting memory clinic have prepared this at home: 100%  To be filled in by me, my care partners, and my clinician during the consultation: 8.3%  To be used by me, my care partners, and my clinician during the consultation: 83.3%  To be used as a reference work: 33.3%  To be used by the person visiting the memory clinic as preparation: 91.7%  To be used as a reference work by the person visiting the memory clinic: 41.7% |
| How much time do you have in your daily practice to offer personal attention to patients, other than the medical aspects? | 0-5 min: 25%  6-10 min: 33.3%  11-15 min: 16.7%  21-25 min: 8.3%  26-30 min: 8.3% |
| Which type of clinician do you think should use such a tool at the memory clinic?** | Neurologist: 83.3%  AIOS Neurology: 75%  ANIOS Neurology: 66.7%  Geriatrician: 75%  AIOS Geriatrics: 75%  ANIOS Geriatrics: 66.7%  Psychiatrist: 50%  AIOS Psychiatry: 50%  ANIOS Psychiatry: 41.7%  Neuropsychologist: 41.7%  Specialized nurse: 66.7%  Nurse: 58.3% |
| What should be the maximum amount of time that the use of the tool should take you as a healthcare provider in talking to a patient and loved one?** | 0-5 min: 75%  6-10 min: 25%  11-15 min: 16.7% |
| Who do you think should be responsible for introducing the tool to people visiting the memory clinic? ** | Primary care physicians: 41.7%  Clinicians at the memory clinic: 58.3%  Front desk employees at the memory clinic: 66.7%  Societal organisations: 25%  Patient organisations: 33.3%  Healthcare insurers: 8.3% |
| Who do you think should be responsible for the maintenance of the tool?** | Primary care physicians: 25%  Clinicians at the memory clinic: 66.7%  Societal organisations: 41.7%  Patient organisations: 41.7%  Healthcare insurers: 8.3% |
| Who do you think should be responsible for the costs of the tool?** | Clinicians at the memory clinic: 8.3%  Societal organisations: 25%  Patient organisations: 16.7%  Healthcare insurers: 75% |
| If you have to make an estimate, what should be the maximum cost of this device per patient?** | 1-5 euro: 75%  6-10 euro: 41.7%  11-15 euro:16.7%  26-30 euro: 16.7% |

**Division based on ^2^. **Multiple answers were possible. AIOS= doctor in specialist training. ANIOS=doctor not in specialist training. ***Physician researcher.*

**Supplement 10: Phase II: explanation of how input by co-researchers, triangulation questionnaire, triangulation PPI focus group session, and co-designers is reflected in the digital and analogue prototype**

The development and design of ‘Helder in Gesprek’ can directly be traced back to the input of phase I and phase II. For instance, we learned from phase I that it is important that the tool should be short, may be used together with others, combines text and visuals, must have a paper version, and contain ‘old-fashioned’ elements, which are alle elements that can be found in this version of the prototype. Moreover, ‘Helder in Gesprek’ contains statements on all main themes identified in phase II that people find important that their clinicians knows about them (e.g. social contacts, leisure, and life phase). The patient journey mapping and overview of information that is already provided by the memory clinic emphasized that the tool should be concise and does not overlap with existing information. Moreover, it highlighted that the tool must be ideally used prior to the consultation at home and during the consultation, as in the waiting room there is already a lot going on, potentially resulting in cognitive overload. At home after the consultation is also not a good option as the window of opportunity is then already passed. Phase II moreover stressed that people visiting the memory clinic and clinicians can prepare properly for a consultation, tailored communication should ideally take place, people in need of care want to come across clearly and worry now that they do not or forget what they want to say, and that people visiting the memory clinic are in control. These are all addressed in the tool or may be facilitated by the tool. Similarly, elements and characteristics from the lo-fi prototypes by co-designers can be traced back in the current prototype (either 1:1 or the underlying rationale).

## Supplement 11: Phase III: UX questionnaire people with cognitive complaints and care partners

**Part 1: Personal questions**

In this section, we will ask you questions about your background, such as your age and education. Please fill in the circle that is correct for you or your situation. There are no right or wrong answers. You can choose 1 answer. Choose the answer that suits you best.

1. **Are you a:**

-
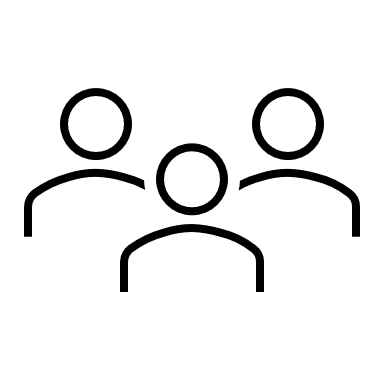
Man
- Woman
- Non-binary person
- Other, namely: ………………………
- I prefer not to tell.

1. **Please fill in your age.**

……………years

1. **Which school or program did you last complete with a degree?**
   - None or elementary school
   - Primary or preparatory vocational education
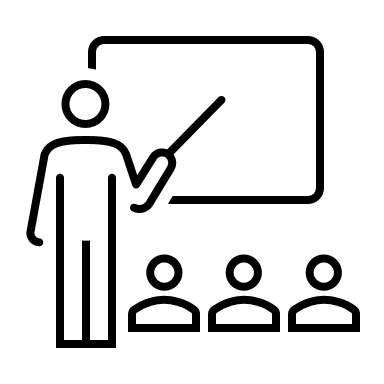
(vso, vmbo-b, vmbo-k, vmbo-g, vmbo-t, mavo, mulo, lts, leao, lhno of meao)
   - Lower grades of secondary school of HAVO or VWO
   - Assistant training (mbo-1)
   - Higher grades of secondary school of HAVO, VWO, HBS, or MMS
   - Basic vocational training (mbo-2)
   - Vocational training (mbo-3)
   - Middle management and specialist education (mbo-4)
   - Higher vocational education (hbo, heao, or hts)
   - University

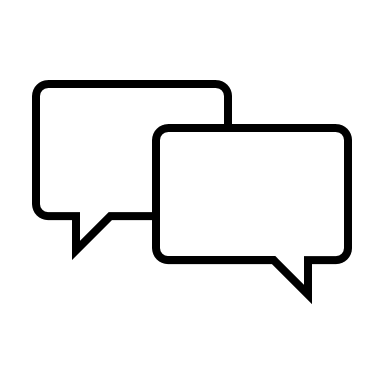

2. **Has a healthcare professional ever told you that you…**

- Have dementia
- Have Mild Cognitive Impairment (MCI)
- Have Subjective Cognitive Decline (SCD)
- A healthcare professional has not told me this.

1. **What language or languages do you speak at home?**

………………………………………………………………………………

1. **In what country were you born?**

………………………………………………………………………………

1. **What digital devices do you use at home?**Digital devices are devices that use technology, such as a smartphone, laptop, computer, or tablet. You may choose several answers to this question.

- A smartphone
  - Android smartphone
  - Apple smartphone
- A laptop or desktop computer
- A tablet
- I do not use digital devices.
- Other, namely: ……………………………………………………

This is the end of Part 1. You may stop completing it here. You will now start testing ‘Helder in Gesprek’.

**Part 2: Questions about ‘Helder in Gesprek’**

In this section, we ask you questions about the ‘Helder in Gesprek’ tool. Please fill in the circle that is correct for you or your situation. There are no right or wrong answers. You can choose 1 answer. Choose the answer that suits you best.

1. **‘Helder in Gesprek’ would help me more quickly mention topics that I think are important to discuss with my healthcare professional in the memory clinic.**
   - Very likely
   - Somewhat likely
   - Neither
   - Somewhat unlikely
   - Not at all likely
2. **With ‘Helder in Gesprek’ I could have better conversations with clinicians in the memory clinic.**
   - Very likely
   - Somewhat likely
   - Neither
   - Somewhat unlikely
   - Not at all likely
3. **‘Helder in Gesprek’ would ensure that I discuss more topics with clinicians in the memory clinic that are important to me.**
   - Very likely
   - Somewhat likely
   - Neither
   - Somewhat unlikely
   - Not at all likely
4. **Using ‘Helder in Gesprek’ would help me better indicate what is important to me during the conversation with a healthcare professional in the memory clinic.**
   - Very likely
   - Somewhat likely
   - Neither
   - Somewhat unlikely
   - Not at all likely
5. **‘Helder in Gesprek’ would make conversations with a healthcare professional in the memory clinic easier.**
   - Very likely
   - Somewhat likely
   - Neither
   - Somewhat unlikely
   - Not at all likely
6. **I think ‘Helder in Gesprek’ is useful for my conversation at the memory clinic.**
   - Very likely
   - Somewhat likely
   - Neither
   - Somewhat unlikely
   - Not at all likely
7. **I could quickly learn how to use ‘Helder in Gesprek’.**
   - Very likely
   - Somewhat likely
   - Neither
   - Somewhat unlikely
   - Not at all likely
8. **The use of ‘Helder in Gesprek’ would be clear and understandable.**
   - Very likely
   - Somewhat likely
   - Neither
   - Somewhat unlikely
   - Not at all likely
9. **I**  **would quickly become proficient in using ‘Helder in Gesprek’.**
   - Very likely
   - Somewhat likely
   - Neither
   - Somewhat unlikely
   - Not at all likely
10. **I could easily use ‘Helder in Gesprek’.**
    - Very likely
    - Somewhat likely
    - Neither
    - Somewhat unlikely
    - Not at all likely
11. **I plan to use ‘Helder in Gesprek’ in the future during conversations with my healthcare professional in the memory clinic.**
    - Very likely
    - Somewhat likely
    - Neither
    - Somewhat unlikely
    - Not at all likely
12. **I will try to use ‘Helder in Gesprek’ during conversations with my healthcare professional in the memory clinic.**
    - Very likely
    - Somewhat likely
    - Neither
    - Somewhat unlikely
    - Not at all likely
13. **How likely would you be to recommend ‘Helder in Gesprek’ to someone who has an appointment at the memory clinic?***Circle the number that is right for you. 0= not at all likely and 10= very likely.*

| **0** | **1** | **2** | **3** | **4** | **5** | **6** | **7** | **8** | **9** | **10** |
| --- | --- | --- | --- | --- | --- | --- | --- | --- | --- | --- |

You have completed the second part of the questionnaire! We are happy about that. On the next page, the third part begins. You can start right away.

**Part 3: Digital devices and information**

Now we will ask you questions about information seeking and understanding. Please fill in the circle that is correct for you or your situation. There are no right or wrong answers. You can choose 1 answer. Choose the answer that suits you best.

1.
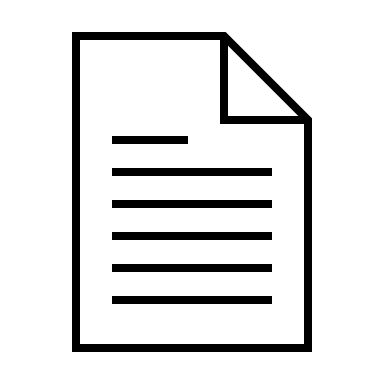
**When you receive leaflets, letters or brochures from your GP, hospital or pharmacy, how often does it happen that the letters are too small to read even if you wear glasses?**

- Never
- Almost never
- Sometimes
- Often

1. **When you receive leaflets, letters or inserts from your GP, hospital or pharmacy, how often do you find yourself reading words in inserts, letters or leaflets that you do not understand?**

- Never
- Almost never
- Sometimes
- Often

1. **When you receive leaflets, letters or brochures from your GP, hospital or pharmacy, how often do you find that you have to re-read sentences to understand them?**

- Never
- Almost never
- Sometimes
- Often

1. **If you get leaflets, letters or inserts from your GP, hospital or pharmacy, how often do you need someone to help you read and understand them?**

- Never
- Almost never
- Sometimes
- Often


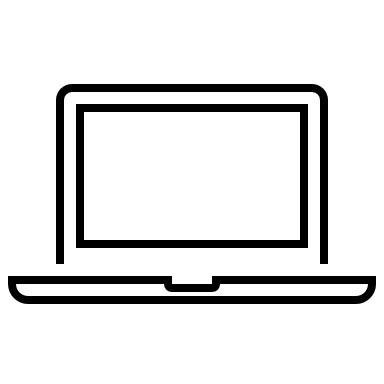

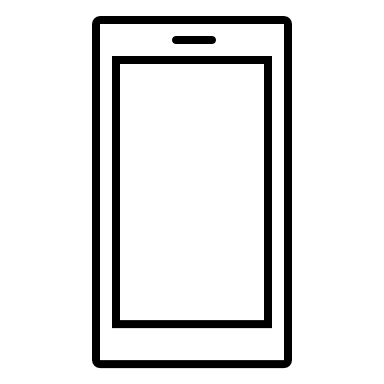
**
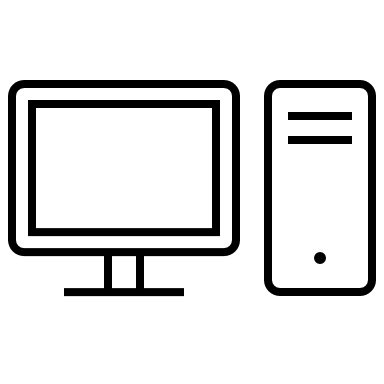
**There is a lot of health and disease information available on the internet these days. The following questions are about whether you use this. We also ask you how difficult or easy you find it to use the internet for your health. Please fill the circle that is correct for you or your situation. There are no right or wrong answers. You can choose 1 answer. Choose the answer that suits you best

1. **If you use a computer, how easy or difficult do you find it to use (type on) the keyboard?**

- Very easy
- Quite easy
- Quite difficult
- Very difficult

1. **If you use a computer, how easy or difficult do you find it to use the mouse (e.g. to move the cursor to the right place or click)?**

- Very easy
- Quite easy
- Quite difficult
- Very difficult

1. **If you use a computer, how easy or difficult do you find it to use the ‘buttons’ and ‘references’ (links or hyperlinks) on websites?**

- Very easy
- Quite easy
- Quite difficult
- Very difficult

1. **When you search the internet for information about health, how easy or difficult do you find it to choose from the information you find?**

- Very easy
- Quite easy
- Quite difficult
- Very difficult

1. **When searching the internet for health-related information, how easy or difficult do you find it to type the right words/search terms into the search engine to quickly find the right information (e.g. via Google)?**

- Very easy
- Quite easy
- Quite difficult
- Very difficult

1. **When you search the internet for information about health, how easy or difficult do you find it to find exactly what you are looking for?**

- Very easy
- Quite easy
- Quite difficult
- Very difficult

1. **When searching the internet for health-related information, how easy or difficult do you find it to determine whether the information is reliable?**

- Very easy
- Quite easy
- Quite difficult
- Very difficult

1. **When searching the internet for information about health, how easy or difficult do you find it to determine whether the information on a website is written with underlying commercial goals? (e.g. by people who want to sell a product).**

- Very easy
- Quite easy
- Quite difficult
- Very difficult

1. **When searching the internet for health information, how easy or difficult do you find it to look at different websites to check whether they give the same information?**

- Very easy
- Quite easy
- Quite difficult
- Very difficult

1. **When you search the internet for information about health, how easy or difficult do you find it to determine whether the information found applies to you?**

- Very easy
- Quite easy
- Quite difficult
- Very difficult

1. **When searching the internet for health-related information, how easy or difficult do you find it to use/apply the information found in your daily life?**

- Very easy
- Quite easy
- Quite difficult
- Very difficult

1. **When searching the internet for information about health, how easy or difficult do you find it to make good decisions about your health with the information you find? (e.g. about diet, medication or whether or not to consult a doctor)**

- Very easy
- Quite easy
- Quite difficult
- Very difficult

1. **When you search the internet for health information, how often does it happen that you get lost on a website or the internet?**

- Never
- Sometimes
- Frequently
- Often

1. **When searching the internet for health-related information, how often does it happen that you do not remember how to go back to a previous page?**

- Never
- Sometimes
- Frequently
- Often

1. **When you search the internet for information about health, how often does it happen that you click on something, and you get to see something completely different from what you expected?**

- Never
- Sometimes
- Frequently
- Often

1. **How difficult or easy do you find it to clearly express your questions or concerns about your health in a message or email (e.g. to your doctor, on a forum or on Facebook)?**

- Very easy
- Quite easy
- Quite difficult
- Very difficult

1. **How difficult or easy do you find it to properly express your opinions, thoughts or feelings in writing in a message or email (e.g. to your doctor, on a forum or on Facebook)?**

- Very easy
- Quite easy
- Quite difficult
- Very difficult

1. **In a message or email (e.g. to your doctor, on a forum or on Facebook), how difficult or easy do you find it to write your message in such a way that people understand exactly what you mean?**

- Very easy
- Quite easy
- Quite difficult
- Very difficult

You only need to complete the questions below (43 to 45) if you have ever posted on social media such as Facebook or Twitter, a public forum, or a (healthcare) review site, such as Zorgkaart Nederland. Does this not apply to you? Then you can skip this question and go on to question 46 on the next page.

1. **If you post on a public forum or social media (such as Facebook and Twitter), how often do you find it difficult to judge who can read your post?**

- Never
- Sometimes
- Frequently
- Often

1. **If you post on a public forum or social media (such as Facebook and Twitter), how often do you (intentionally or unintentionally) put privacy-sensitive information about yourself in your post? (such as your name or address)**

- Never
- Sometimes
- Frequently
- Often

1. **If you post a message on a public forum or social media (such as Facebook and Twitter), how often do you (intentionally or unintentionally) put privacy-sensitive information of people close to you in your message?**

- Never
- Sometimes
- Frequently
- Often

1. **Do you have any questions or remarks about this questionnaire?**
   *For example, about the questionnaire itself. This question is not mandatory.*


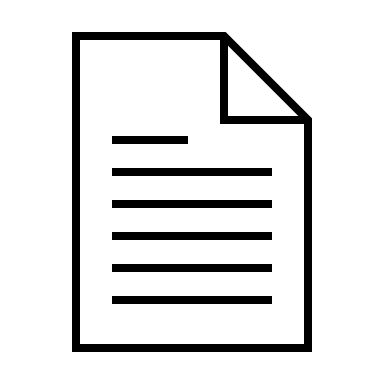
…………………………………………………………………………………………………………………………………………………………………………………………………………………………………………………………………………………………………………………………………………………………………………………………………………

1. **Do you want to know the results of this study?***If you wish, we will send you a summary of the results of the study via an e-mail or by post.*

- Yes, I would like to receive a summary. My e-mail or postal address is:

…………………………………………………………………………………

- No, I prefer not to.

## Supplement 12: Phase III: UX questionnaire clinicians

**Part 1: Personal questions**

In this section, we will ask you questions about your background, such as your age and education. Please fill in the circle that is correct for you or your situation. There are no right or wrong answers. You can choose 1 answer. Choose the answer that suits you best.

1. **Are you a:**

-
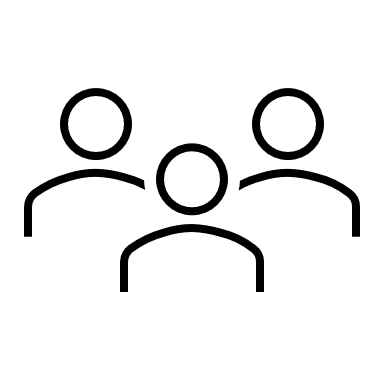
Man
- Woman
- Non-binary person
- Other, namely: ………………………
- I prefer not to tell.

1. **Please fill in your age.**

……………years

1. **Which school or program did you last complete with a degree?**
   - None or elementary school
   - Primary or preparatory vocational education
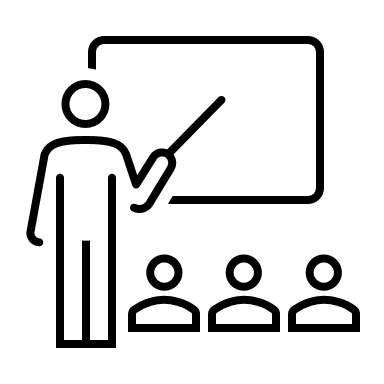
(vso, vmbo-b, vmbo-k, vmbo-g, vmbo-t, mavo, mulo, lts, leao, lhno of meao)
   - Lower grades of secondary school of HAVO or VWO
   - Assistant training (mbo-1)
   - Higher grades of secondary school of HAVO, VWO, HBS, or MMS
   - Basic vocational training (mbo-2)
   - Vocational training (mbo-3)
   - Middle management and specialist education (mbo-4)
   - Higher vocational education (hbo, heao, or hts)
   - University

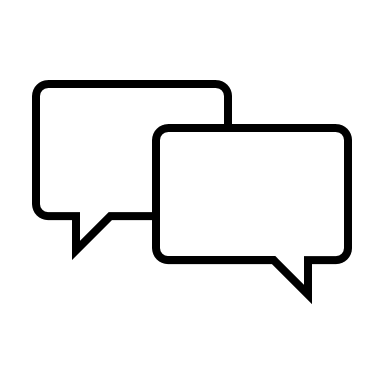

2. **What is your profession in the memory clinic?**

- Neurologist
- AIOS neurology
- ANIOS neurology
- Geriatrician
- AIOS geriatrics
- ANIOS geriatrics
- Psychiatrist
- AIOS psychiatry
- ANIOS psychiatry
- Neuropsychologist
- Front desk employee
- Medical doctor not in specialist training [NL: basisarts]
- Specialised nurse
- Nurse
- Casemanager
- Occupational therapist
- Physician assistant
- Social worker
- Other, namely: …

1. **How many years of work experience do you have in the memory clinic?**
   - 0-10 years
   - 11-20 years
   - 21-30 years
   - 31-40 years
   - 41-50 years
   - More than 50 years
   - Other, namely: …
2. **What language or languages do you speak at home?**

………………………………………………………………………………

1. **In what country were you born?**

………………………………………………………………………………

1. **What digital devices do you use at home?**Digital devices are devices that use technology, such as a smartphone, laptop, computer, or tablet. You may choose several answers to this question.

- A smartphone
  - Android smartphone
  - Apple smartphone
- A laptop or desktop computer
- A tablet
- I do not use digital devices.
- Other, namely: ……………………………………………………

This is the end of Part 1. You may stop completing it here. You will now start testing ‘Helder in Gesprek’.

**Part 2: Questions about ‘Helder in Gesprek’**

In this section, we ask you questions about the ‘Helder in Gesprek’ tool. Please fill in the circle that is correct for you or your situation. There are no right or wrong answers. You can choose 1 answer. Choose the answer that suits you best.

1. **Please answer the following statements on ‘Helder in Gesprek’.***Put a cross in the box that is right for you.*

|  | **Very likely** | **A bit likely** | **Neither** | **A bit unlikely** | **Very unlikely** |
| --- | --- | --- | --- | --- | --- |
| **‘**Helder in Gesprek’ would help me to get to know my patients **faster.** |  |  |  |  |  |
| ‘Helder in Gesprek’ would help me provide **better care** to my patients. |  |  |  |  |  |
| ‘Helder in Gesprek’ would make my consultation more **efficient.** |  |  |  |  |  |
| By using ‘Helder in Gesprek’, I would be able to get to know my patients **better.** |  |  |  |  |  |
| ‘Helder in Gesprek’ would make it **easier** to get to know my patients better. |  |  |  |  |  |
| I think ‘Helder in Gesprek’ would be **helpful** in getting to know my patients. |  |  |  |  |  |
| I could **quickly learn** how to use ‘Helder in Gesprek’. |  |  |  |  |  |
| Working with ‘Helder in Gesprek’ **during consultations** with patients would be **clear and understandable**. |  |  |  |  |  |
| I would **quickly become proficient** in using ‘Helder in Gesprek’ **during conversations** with patients. |  |  |  |  |  |
| I would be able to use ‘Helder in Gesprek’ **easily.** |  |  |  |  |  |
|  | **Totally agree** | **Agree** | **Neither agree nor disagree** | **Disagree** | **Totally disagree** |
| I **plan to use** ‘Helder in Gesprek’ in my consultations in the future. |  |  |  |  |  |
| I **will try to use** ‘Helder in Gesprek’ during my consultations. |  |  |  |  |  |

1. **How likely would you be to recommend ‘Helder in Gesprek’ to someone who has an appointment at the memory clinic?***Circle the number that is right for you. 0= not at all likely and 10= very likely.*

| **0** | **1** | **2** | **3** | **4** | **5** | **6** | **7** | **8** | **9** | **10** |
| --- | --- | --- | --- | --- | --- | --- | --- | --- | --- | --- |

1. **How likely would you be to recommend ‘Helder in Gesprek’ to a colleague at the memory clinic?***Circle the number that is right for you. 0= not at all likely and 10= very likely.*

| **0** | **1** | **2** | **3** | **4** | **5** | **6** | **7** | **8** | **9** | **10** |
| --- | --- | --- | --- | --- | --- | --- | --- | --- | --- | --- |

You have completed the second part of the questionnaire! We are happy about that. On the next page, the third part begins. You can start right away.

**Part 3: Digital devices and information**

There is a lot of information about health and illness available on the internet these days. The following questions are about whether you use this. We also ask you how difficult or easy you find it to use the internet for your health. Please circle the number that is right for you.

How do you rate your own digital skills?

Please circle the number that is right for you.

0= not good at all and 10= very good

1. **How do you rate your own digital skills?***Circle the number that is right for you. 0= not good at all and 10= very good.*

| **0** | **1** | **2** | **3** | **4** | **5** | **6** | **7** | **8** | **9** | **10** |
| --- | --- | --- | --- | --- | --- | --- | --- | --- | --- | --- |

1. **Do you have any questions or remarks about this questionnaire?**
   *For example, about the questionnaire itself. This question is not mandatory.*


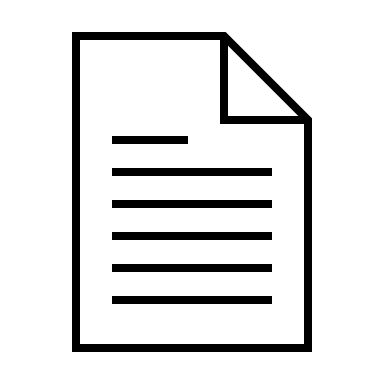
…………………………………………………………………………………………………………………………………………………………………………………………………………………………………………………………………………………………………………………………………………………………………………………………………………

1. **Do you want to know the results of this study?***If you wish, we will send you a summary of the results of the study via an e-mail or by post.*

- Yes, I would like to receive a summary. My e-mail or postal address is:

…………………………………………………………………………………

- No, I prefer not to.

**Supplement 13: Phase III: topic guide UX focus groups**

1. What do you think of the goal of ‘Helder in Gesprek’?
2. What [PrEmo] puppet best reflects your opinion on ‘Helder in Gesprek’? And why?
3. Please answer the following statements:
   1. I think that ‘Helder in Gesprek’ is a useful tool for me: yes, a bit yes and a bit no, or no. And why?
   2. I think that ‘Helder in Gesprek’ is easy to use for me: yes, a bit yes and a bit no, or no. And why?
   3. I plan to use ‘Helder in Gesprek’ during a consultation in the memory clinic: yes, a bit yes and a bit no, or no. And why?
4. How can we improve ‘Helder in Gesprek’?
   1. What do you think about the lay-out?
   2. What do you think about the location of certain information?
   3. Do you miss any information?

**Supplement 14: Phase III: Usability issues encountered in the digital prototype.**

The results demonstrate that participants did not find the navigation to be clear or intuitive (see supplementary table 4). The participant was unable to locate the required page, which caused confusion and frustration. Participants also struggled due to incomplete instructions containing terms that did not correspond to the required activity, or words that were extremely similar and could be misinterpreted. Language was a problem with both the digital prototype's instructions and the statements and response choices. For instance, participants experienced confusion and misunderstanding when abstract notions, adjectives, homonyms, jargon and negation were employed. Furthermore, the participants found the statements and questions asked to be confusing, which resulted in unanswered questions. Furthermore, star ratings indicating the statement's significance or the participant's willingness to discuss the issue with a healthcare professional were frequently omitted. It was noted that the background and the star rating bar were not sufficiently contrasting, which meant that participants did not notice this element. Participants also encountered difficulty differentiating between clickable and non-clickable items, which led to frequent attempts to click on non-clickable elements. The absence of system feedback was another perception problem that frequently resulted in unintentional behaviours. Following the submission of responses to a question, participants receive minimal feedback, which prompts them to select questions that have already been answered and, in certain instances, to re-answer the question. Additionally, to obtain the feedback, the user must reopen the pop-up screen containing the question and answers. This interruption to the participants' question-and-answer session caused confusion and irritation. Furthermore, participants frequently required multiple attempts to successfully click a button. Additionally, changes in clicking technique (e.g. increased pressure or duration) inadvertently resulted in scrolling or selection of text on a button.

**Supplementary Table 4. Usability issues encountered in the digital prototype**

| Specification | Problem | Frequency of problem encountered | Violated DEMIGNED principle |
| --- | --- | --- | --- |
| Theme 1: Perception | | | |
| Subtheme: Minimal system feedback | | | |
| Statement questions | After answering a question, there is not enough feedback that the question has been answered and how it was answered. This causes users to repeatedly click on already answered question and in some cases re-answer the question. | 24 | P - System feedback  C - Progress |
| Number of answered statements | On the overview page minimal feedback is provided about the amount of statements already filled in and to be filled in. This causes the user to not know how to continue to the next step when not all statements are filled in yet. | 4 | P - System feedback  C - Progress |
| Empty text field | When the text field for name and surname is not filled in and the user would like to send the summary, minimal feedback is provided that the field should be filled in, causing the user to think that they have already sent the summary while this is not the case. | 1 | P - System feedback |
| Subtheme: Distinguishable elements | | | |
| Clickable elements | There is no clear distinction between clickable elements and non-clickable elements, causing users to repeatedly try to click non-clickable elements. | 26 | P - Clickable areas  C - Icons |
| Star rating | The colour of the stars in the star rating bar does not contrast enough with the background. This causes the users to overlook the star rating. | 11 | P - Distinguishable colours |
| Closing pop-up screen | The user tries to scroll through the answers on the pop-up screen, but does this next to the pop-up screen which causes the pop-up screen to close. | 2 | P - Distinguishable colours  P - Compartmentalized UIs |
| Statement and answer buttons | The user scrolls to the next statement without the answer buttons visible yet. As the answer buttons of the last statement are still visible, the user answers the next statement by using the buttons belonging to the last statement. | 1 | P - Compartmentalized UIs |
| Theme 2: Speech and Language | | | |
| Subtheme: Understandability | | | |
| Negation | The statement 'I do not work anymore' is confusing for the user due to the negation, which creates uncertainty about which answer to select. | 7 | S - Understandability |
| Jargon | The answer option with 'dementia' does not resonate with the user, as the user is more familiar with 'Alzheimer's disease', which creates uncertainty about which answer to select. | 1 | S - Understandability  F - Content |
| Adjectives | The use of adjectives such as 'regularly' or 'large' creates uncertainty in the user about the applicability of the statement or answer option. | 2 | S - Understandability |
| Homonyms | The use of words with different meanings causes confusion for the user about the interpretation of the answer option. | 1 | S - Understandability |
| Abstract concepts | The use of abstract concepts, such as 'the future' or 'complaints', in statements, causes uncertainty about how to answer the statement, as the user finds it difficult to place the statements in context. This can lead to users giving the incorrect answer. | 12 | S - Understandability  F - Content |
| Double statement | The statement 'I work/I do volunteer work' causes confusion, as the user does not know how to answer the statement when only one of the two applies. | 1 | S - Understandability |
| Redundant statements | Opposing statements cause confusion as the answer of the one statement directly implies the answer of the other statement. | 3 | S - Understandability |
| Subtheme: Text input | | | |
| Answer option 'else' | When the user selects the answer option 'else', they expect a free text field to explain their answer. | 6 | S - Input |
| Subtheme: Similarity | | | |
| Similarity questions | The questions asked for each statement are very similar and the question asked per statement look very similar, causing users to not remember whether they already answered the questions, which causes confusion and questions to remain unanswered. | 14 | S - Understandability |
| Theme 3: Frame of Mind | | | |
| Subtheme: Limited answer options | | | |
| Statements | There are no other answer options available for the statements other than 'yes' and 'no'. However, the user may feel that their answer lies somewhere in between, which creates uncertainty about how to answer the statement. | 7 | F - Content |
| Question physical exercise | The user has the option to choose between 'weekly', 'monthly', and 'yearly' to indicate the frequency of physical exercise. The user misses the option for 'daily'. | 4 | F - Content |
| Why discuss this topic | The user misses the option 'I do not want to discuss this topic' as answer option when being asked 'why would you like to discuss this topic with a healthcare provider?'. The absence of this answer option potentially makes user choose an inapplicable answer. | 9 | F - Content |
| Star rating | The lowest score possible on the star rating scale is 1 (as, when the first star is selected, it is not possible to deselect the star). The user would like to be able to give 0 stars. | 2 | F - Content  F - Support |
| Subtheme: Appropriate content | | | |
| Answer options | The answer options for the question 'why is this important for you' are the same for all statements. However, these answer options are positively phrased, which is not a good fit with all statements. This can cause frustration to the user and the user might feel misunderstood regarding the seriousness of the situation or experience. | 9 | F - Content |
| Question following star rating | After indicating that discussing the topic with the healthcare provider is not desirable by selecting 1 or just few stars, the user is still asked why they would like to discuss the topic. This causes frustration to the user. | 5 | F - Content |
| Star rating | The number of stars selected to indicate the desire to discuss the topic with the healthcare provider does not match with the answer voiced by the user or the user voices not to know how to use the star rating. This might be caused by misunderstanding of the concept of star rating. | 6 | F - Content |
| Multiple choice | The user finds multiple answer options applicable, but does not select all answers as this feels inappropriate to them. | 1 | F - Content  C - Instructions |
| Summary receiver | It is not explicitly mentioned to whom the summary is sent. The user does not feel comfortable sending their information to a healthcare professional when it is not known who this is, due to privacy reasons. | 1 | F - App settings |
| Theme 4: Cognition | | | |
| Subtheme: Navigation | | | |
| Expectations buttons | Clicking the 'home' button or 'question mark' button often does not yield the expected result for the user. This causes frustration and confusion for the user. | 16 | C - Navigation  C - Icons;  F - Support |
| To next statement | After answering the first statement, the user does not know how to navigate to the next statement. | 5 | C - Navigation |
| Drop down menu | The user expects a drop down menu on the top right of the screen to easily navigate through the different steps. | 2 | C - Navigation  C - Functionalities  C - Icons |
| Navigation through the steps | The user is unsure about the navigation through the different steps of answering statements, adjusting answers, giving explanations, and making a summary. This causes frustration and confusion to some users, as they feel they are stuck or are not able to reach the page they have in mind. | 21 | C - Navigation  F - Support |
| Results page step 1 | After answering all statements with 'yes' or 'no', the user is being navigated to the 'results step 1' page. The function of this page is unclear to the user and the user expects to be able to adjust their answers or to give explanation to the statements on this page. This causes confusion. | 6 | C - Navigation  C - Instructions |
| Automatically navigated upwards | When returning to the overview page or to the overview page of statements to be explained, the user is navigated to the top of the page. This is confusing when the user was already further in the process, as they now have to scroll to find the right place on the page to continue their action. | 2 | C - Navigation  C - Progress |
| Subtheme: Instructions | | | |
| Minimum and maximum answers | The instruction about a minimum or maximum amount of statements to explain or select are misinterpreted by the user. This might be partly caused by the fact that both instructions include '4 statements', making it easy to confuse the words 'maximum' and 'minimum'. Moreover, the words can be overlooked, in which case the user interprets the instructions as 'exactly 4 statements'. | 5 | C - Instructions  S - Understandability |
| Save and continue | The instruction on the bottom of page 1 explains to 'save and continue' when all statements are answered. However, the button only says 'continue'. This confuses the user, as they do not know how to 'save' their answers. | 2 | C - Instructions  S - Understandability |
| Required to answer | It is not clear to the user whether all statements must be answered and whether it is required to answer all statement questions. This problem comes up when the answer options are not appropriate or when it is unclear to the user whether it is possible to continue when a question has not been answered. | 6 | C - Instructions  F - Content  C - Navigation |
| Summary | It is unclear to the user what the summary entails or how to make the summary. | 5 | C - Instructions |
| Select applicable answer | The user selects the answers that do not apply. This might be due to unclear instructions about when to select an answer. | 1 | C - Instructions; |
| Subtheme: Cognitive overload | | | |
| Home page | The user forgets the task when they end up at the home page and start reading the content. | 3 | C - Instructions  C - Icons |
| Questions statement | The user forgets the statement while answering the question relating to that statement. | 6 | C - Instructions |
| Navigation | The user voices to struggle with the navigation through the steps due to the overload of information on all pages. | 1 | C - Instructions  C - Navigation |
| Subtheme: Icons | | | |
| Statement icon | The icon presented with the statement may cause confusion to the user due to its unclear interpretability. | 3 | C - Icons |
| Text bubble | The orange pictogram that represents the bottom of a text bubble is being interpretated as an arrow. This confuses the user. | 1 | C - Icons |
| Theme 5: Screen sensitivity | | | |
| Clicking button | The user requires multiple attempts to successfully click on a button or element. By adjusting their clicking technique, some users unintentionally scroll or select the text on the button. | 32 | P - Clickable areas  P - Visibility |
| Unintended (momentary) selection | By clicking 'save and continue' on the pop-up screen or while scrolling up and down the page, stars are (momentarily) being selected. | 9 |  |

**Supplement 15: Phase III: Usability issues encountered in the analogue prototype.**

The results of the analogue prototype indicate that the most common issues encountered are related to the visibility of the star rating element, the way statements and questions are interpreted, and the fact that the question "how important is this for you?" has both a free text field and a star rating bar, which may confuse the user (see supplementary table 5). More widespread and common usability issues were caused by cognitive overload, which diverted participants' attention from the TA task at hand, and the incapacity to identify the functions of various page types (such as notes pages or pages meant for a new statement), which in certain cases also resulted in perplexity or annoyance. Additionally, the booklet's structure is directly related to the subsequent issue concerning its future usability. The booklet is bound by two rings that can be opened to change the page order. However, this feature was not widely known among participants, and even those who were aware of it were unable open the rings. Consequently, a misunderstanding arose when the participant was instructed to place statements they wished to discuss with the healthcare provider behind the instruction page. Furthermore, the circumstance caused unhappiness because some participants thought it was necessary to copy the remarks to be discussed with health professionals onto the notes pages.

**Supplementary Table 5. Usability issues encountered in the analogue prototype.**

| Specification | Problem | Frequency of problem encountered | Violated DEMIGNED principle |
| --- | --- | --- | --- |
| Theme 1: Perception | | | |
| Subtheme: Unity | | | |
| Text field 'else' | The text field intended to elucidate the answer option 'else' is being used to further explain other answer options, due to the placement of the lines, which makes it seem as if the lines belong to other answer options as well. | 4 | P - Compartmentalized UIs |
| Questions associated with statement on next page | The user thinks that the questions on the left page refer to the statement on the right page. | 2 | P - Compartmentalized UIs  P - Visibility |
| Subtheme: Distinguishable elements | | | |
| Star rating | The user does not notice the star rating bar or has to take off glasses to recognize the star shapes. | 16 | P - Distinguishable colours  P - Compartmentalized UIs |
| Answer placement | The user writes explanation to statement beneath the orange bar on the front of the statement page. | 1 | P - Compartmentalized UIs  C - Icons |
| Theme 2: Speech and language | | | |
| Subtheme: Interpretability | | | |
| Answer options | The user is not sure whether the answer option is applicable due to the interpretability. | 4 | S - Understandability |
| Statements | The user finds it difficult to answer the statement or questions belonging to statement, due to its (context dependent) interpretability. | 8 | S - Understandability |
| Theme 3: Frame of mind | | | |
| Subtheme: Content | | | |
| Comprehensiveness theme | The user feels that the theme 'future and support' is too short, as there is no statement about the future perspective. | 1 | F - Content |
| Superfluous question | There is a possibility to score the importance of the topic as well as to answer the question on how important the topic is. The user either writes down the answer or fill in the star rating. This superfluity might cause confusion. | 6 | F - Content |
| Selective focus | The user explains that they tend to focus only on the content on the right page, while being aware that there is also content on the left page. | 1 | F - Content |
| Verbal explanation | The user gives verbal explanation to all statements and is not aware of the questions on the back of the statement pages. | 1 | F - Content |
| Theme 4: Cognition | | | |
| Subtheme: Navigation | | | |
| Table of contents | The user expects or wishes for a table of contents in the beginning or at the end of the booklet. | 2 | C - Navigation  F - Support |
| Subtheme: Instructions | | | |
| Instructions placement statements | The user is confused by the instructions in the back of the booklet, that state to 'place the statements' behind the instruction pages. This is mostly due to the fact that it is not clear to the user that the pages can be taken out and relocated in the booklet. | 2 | C - Instructions |
| Unclear instruction | The user does not understand the instruction about the placement of statements answered with 'no', as it was not explained that statements could or should be answered with 'yes' or 'no'. | 1 | C - Instructions |
| Instruction star rating | The user understands the purpose of the star rating after reading the instructions in the back of the booklet. | 1 | C - Instructions |
| Required to answer statements within theme | The user is not sure whether to answer all statements within a theme when the first statement in the theme does not apply. | 1 | C - Instructions |
| Remembering instruction pages | The user feels that the content of the first few pages of the booklet, including instructions and sample answers, should be remembered as they fill in the statements/questions in the booklet. | 2 | C - Instructions  F - Support |
| Subtheme: Cognitive overload | | | |
| Distraction | The user forgets the task while leafing through the instruction pages or through the statements. | 6 | C - Instructions |
| Subtheme: Functionality recognition | | | |
| Extra statements pages | The user thinks that the pages intended for extra statements are to be used for notes. | 1 | C - Instructions |
| Notes pages | The user thinks that the notes pages are to be used for the less important statements. | 1 | C - Instructions |
| New statements | It is unclear to the user where to add new statements in the booklet. The user thinks the free text fields on the back of the statement pages can be used for new statements, or that statements can be added on the front of an already existing statement page. | 5 | C - Instructions |
| Instruction page | The user struggles to identify the instruction page. This is mainly due to the fact that the instruction page is not titled as such, which confuses the user. | 6 | C - Instructions  S - Understandability  F - Support |
| Example cards | It is not clear to the user that the 'example cards' include examples of answers. The pages are being interpreted as instructions, statements to be explained, or answer options that should be used to fill in questions later in the booklet. | 4 | C - Instructions  S - Understandability |
| First statement | It is unclear to the user what the first statement is. | 2 | C - Instructions  P - Compartmentalized UIs |
| Theme | The user finds it difficult to identify the four themes of the statements. | 1 | C - Instructions  P - Visibility |
| Statements to discuss with healthcare professional | The user struggles to find the place to put the statements to discuss with the healthcare professional, or identifies the place for new statements or for notes as the correct location. This causes some confusion and frustration, as some users think they should copy the statements to these pages. | 5 | C - Instructions |
| Extra statement within theme | The user would like to be able to add an extra statement within a theme. | 1 | C - Instructions |
| Theme 5: Booklet anatomy | | | |
| Opening rings | The user is not aware of the possibility of opening the rings or is not able to actually open the rings. | 7 |  |
| Rings | The rings hinder turning pages or get in the way when the user writes down their answer. | 5 |  |
| Pages | The user unintendedly turns two pages at a time or struggles with separating two pages. | 2 |  |
| Digital booklet | The user suggests to answer the questions on their laptop, while struggling with the booklet rings. | 1 |  |
| Thickness booklet | Due to the thickness of the booklet, the user is not sure whether they would go through it before the appointment with the healthcare professional. | 1 | F - Content |

**Supplement 16: Results Product Emotion Measurement Instrument (PrEmo) scores during UX interview and UX focus groups**

**Supplementary Table 6. PrEmo scores results**

| **PrEmo score** | **n=x*** |
| --- | --- |
| 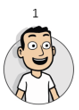 | n=1 |
| 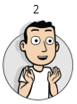 | n=4 |
| 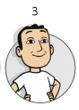 | n=8 |
| 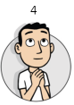 | n=4 |
| 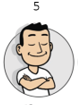 | n=3 |
| 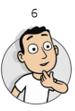 | n=13 |
| 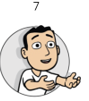 | n=3 |
| 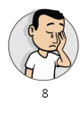 | n=1 |
| 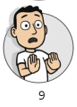 | n=1 |
| 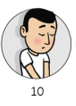 | n=0 |
| 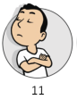 | n=0 |
| 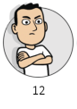 | n=1 |
| 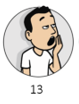 | n=0 |
| 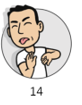 | n=0 |

**participants could choose multiple PrEmo puppets.*

**
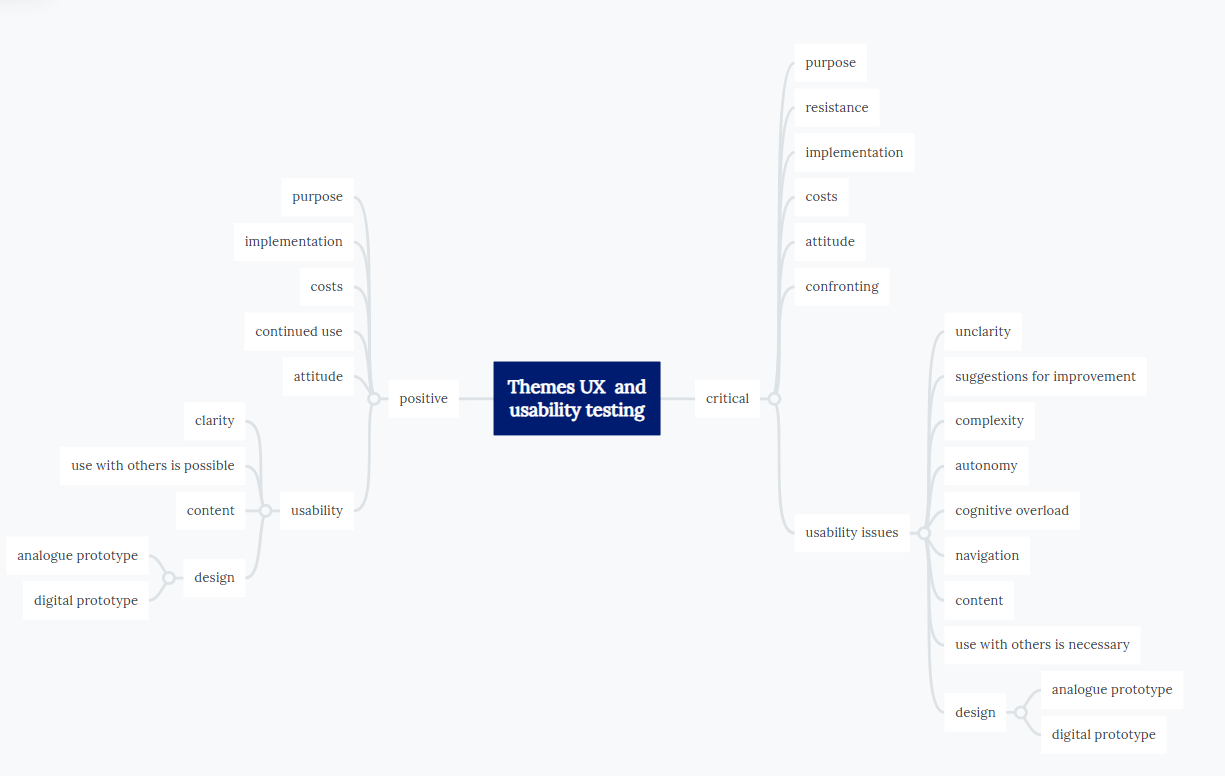
Supplement 17: Themes based on UX interviews and UX focus groups**

**Supplementary Figure 1. Code tree based on UX interviews and UX focus groups.**

**Supplement 18: Phase III: Exemplary quotes UX interviews and UX focus groups**

**Supplementary Box 1. Exemplary quotes UX interviews and UX focus groups**

*‘It does make you think, and later, on the train back home, I'm going to think it over again. And then discuss it with my brother. I'll also forward it to him. Those are things we discuss together.*’ (Usability test 4, person with cognitive complaints, audio ID 66)

*‘Yes, for the reason I just mentioned. I think it's very nice that with the help of this, it can give an overview in a period in which you are overwhelmed with quite a lot, and that it can provide structure but also has flexibility to do what you want and give direction to the things that are important to you at that moment. And at the same time so that a care professional can see that, Can keep an eye on that a little bit, so thinks oh but I do see that you live alone or that you have these things that you don't think are important now either...... But.....I hope a care professional then thinks. Oh, that's important to note at least somewhere if.... Yes there In the future sometime to think about it and ask about it, something like that.’* (Focus group 2, care partners, audio ID 99)

‘*I think it's complicated and If you’ve got dementia, I don't think you can do anything with it Without someone being there to tell you what to do. I may sound a bit like a jerk, but if I almost don't get it already then I think a lot of people would struggle with that … I miss overview. If you've browsed through it a few times, well, well then you see how it's put together. But If you are confronted with it like that then....’* (usability test 14, person with cognitive complaints, audio ID 94)

*‘My first reaction was, oh boy, it* [the book] *is quite thick. But that was because I thought oh, if it's completely filled with content and I have to work through it then....’* (usability test, clinician 7, audio ID 89)

## References for supplementary materials

1. Kochanowska M and Gagliardi WR. The double diamond model: In pursuit of simplicity and flexibility. *Perspectives on Design II: Research, Education and Practice* 2022: 19-32.

2. Pleijers A, & De Vries, R. . 3. Indeling van opleiding op basis van niveau en oriëntatie.: Centraal Bureau voor de Statistiek, 2021.
