# Supplementary material for: Design and development of ‘Helder in Gesprek’: A tool to support person-centred communication in memory clinics
Source: Digit Health. 2026 Jan 20;12:20552076251412631. doi: 10.1177/20552076251412631 (PMC12820018; doi:10.1177/20552076251412631)
Supplement: sj-pdf-2-dhj-10.1177_20552076251412631 - Supplemental material for Design and development of ‘Helder in Gesprek’: A tool to support person-centred communication in memory clinics [file sj-pdf-2-dhj-10.1177_20552076251412631.pdf]

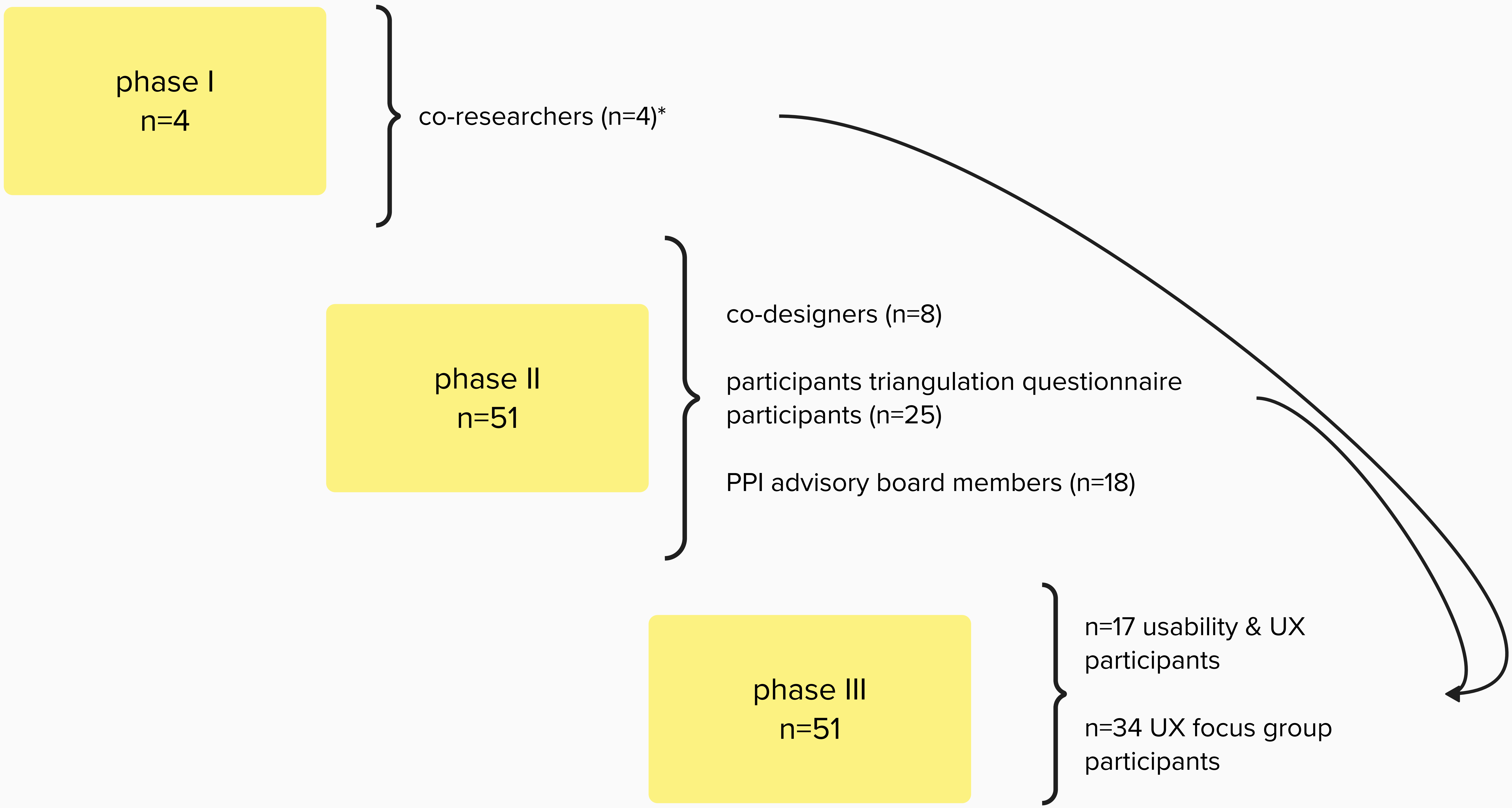
phase I
n=4
co-researchers (n=4)*
phase II
n=51
co-designers (n=8)
participants triangulation questionnaire participants (n=25)
PPI advisory board members (n=18)
phase III
n=51
n=17 usability & UX participants
n=34 UX focus group participants
